# Tailoring Cell Behavior and Antibacterial Properties on Zirconia Biomaterials through Femtosecond Laser-Induced Micropatterns and Nanotopography

**DOI:** 10.1021/acsami.4c22433

**Published:** 2025-05-10

**Authors:** Nerea Garcia-de-Albeniz, Daniel W. Müller, Frank Mücklich, Maria-Pau Ginebra, Emilio Jiménez-Piqué, Carlos Mas-Moruno

**Affiliations:** † Center for Structural Integrity, Reliability and Micromechanics of Materials (CIEFMA), Department of Materials Science and Engineering, Universitat Politècnica de Catalunya. BarcelonaTech (UPC), Av. Eduard Maristany, 16, Barcelona 08019, Spain; ‡ Biomaterials, Biomechanics and Tissue Engineering Group (BBT), Department of Materials Science and Engineering, Universitat Politècnica de Catalunya. BarcelonaTech (UPC), Av. Eduard Maristany, 16, Barcelona 08019, Spain; § Barcelona Research Center in Multiscale Science and Engineering, Universitat Politècnica de Catalunya. BarcelonaTech (UPC), Av. Eduard Maristany, 16, Barcelona 08019, Spain; ∥ Functional Materials, Department of Materials Science and Engineering, 9379 Saarland University, Saarbrücken 66123, Germany; ⊥ Centro de Investigación Biomédica en Red—Bioingeniería, Biomedicina y Nanomedicina (CIBER-BBN), Instituto de Salud Carlos III, Madrid 28029, Spain; # Institute for Bioengineering of Catalonia (IBEC), Barcelona Institute of Science and Technology, Barcelona 08028, Spain

**Keywords:** zirconia, dental implants, laser patterning, topography, osteointegration, antibacterial properties

## Abstract

This study explores the potential of ultrashort pulsed-direct laser interference patterning (USP-DLIP) to fabricate micropatterns on zirconia surfaces, aimed at enhancing their cell-instructive and antibacterial properties for biomedical applications. A femtosecond laser was employed to fabricate 3 and 10 μm periodic linear (L3 and L10) and grid (G3 and G10) patterns on tetragonal zirconia polycrystal stabilized with 3% molar yttrium oxide (3Y-TZP). The patterns exhibited homogeneous, high-aspect-ratio structures with laser-induced nanotopography within the grooves while maintaining minimal surface damage. All patterns significantly enhanced human mesenchymal stem cell (hMSCs) adhesion, spreading, and migration through topographical guidance and nanotopography-induced cell anchoring. Pattern geometry influenced cell morphology and migration: linear patterns induced high elongation and alignment along the grooves, leading to unidirectional migration, while grid structures promoted widespread cells with bidirectional alignment, promoting bidirectional migration. Antibacterial assessment using Pseudomonas aeruginosa (P. aeruginosa) (Gram-negative) and Staphylococcus aureus (S. aureus) (Gram-positive) revealed a size-dependent bacterial response. The patterns of lower periodicity (L3 and G3) showed superior antibacterial properties, reducing bacterial colonization through distinct mechanisms: mechanical trapping for P. aeruginosa (25% reduction) and disruption of bacterial aggregation for S. aureus (30% reduction). Coculture experiments with hMSCs and bacteria confirmed that L3 and G3 surfaces effectively combined enhanced cell adhesion with reduced bacterial colonization, highlighting the potential of USP-DLIP for developing multifunctional cell-instructive and antibacterial biomaterial surfaces.

## Graphical abstract

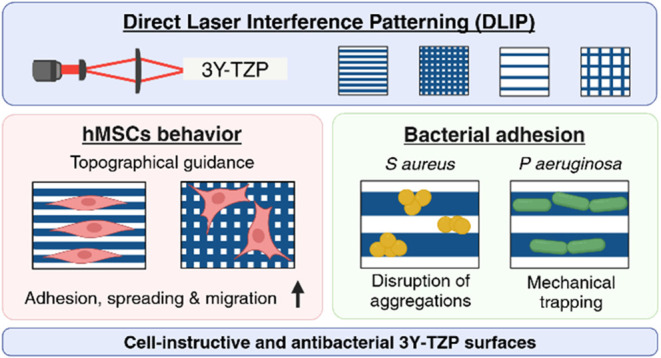

## 1 Introduction

Tetragonal zirconia polycrystal stabilized with 3% molar yttrium oxide (also denoted as 3Y-TZP) is an excellent biomaterial to produce dental implants owing to its unique combination of high mechanical strength and corrosion resistance, optimal biocompatibility, and low plaque adhesion.^1^ Additionally, this material is increasingly replacing traditional titanium implants, offering superior esthetic advantages (i.e., natural tooth-like color)^2^ and reducing the concerns related to metal allergies.^3,4^

The clinical success of a dental implant depends on its ability to achieve optimal and long-lasting osseointegration with the surrounding bone tissue.^5^ This process begins with protein absorption onto the implant surface, followed by host cell attachment and differentiation, and subsequent bone matrix deposition, thereby establishing a functional and structural bone-implant connection.^6^ However, bacterial infections are a critical challenge as they can impair the osseointegration and compromise long-term stability of the implant.^7^ Indeed, the fate of the implant is determined by a competitive process between the host tissue integration and the bacterial colonization, widely known as the “race for the surface”.^8^ Consequently, to ensure implant success, the surface must simultaneously promote strong cell adhesion while preventing bacterial attachment, which could otherwise lead to biofilm formation and peri-implantitis.

In this context, the surface properties of the implant, particularly its surface chemistry, topography and wettability, are crucial in effectively driving the bone healing process.^9^ Among these, surface topography (i.e., surface roughness) is a determinant in promoting successful osseointegration. Indeed, a large number of in vivo studies reported improved bone-to-implant contact (BIC) on rough zirconia when compared to nonmodified surfaces.^10–12^ At the cellular level, these positive outcomes are directly linked to enhanced values of adhesion, migration, proliferation and differentiation of human mesenchymal stem cells (hMSCs) on the modified surfaces.^13–15^ Such beneficial effects on cell behavior are generally attributed to the higher specific surface area for protein adsorption and increased anchoring points for cell attachment, available on surfaces with micrometer and submicrometer roughness values, compared to smoother substrates. Complementary, nanoscale roughness promotes the formation of filopodia and focal adhesions, which strengthens cell anchoring and differentiation.^16^ Moreover, specific topographical features at both the micro- and nanoscale can also guide cell behavior through a phenomenon known as “contact guidance”, a process in which topographical cues activate specific signaling pathways that reorganize the cytoskeleton, alter cell morphology, and regulate cell functions.^9,17,18^ For instance, nanodisordered topographical cues^19^ and nanogrid structures^20^ can effectively guide the osteogenic differentiation of hMSCs. Similarly, microscale features, such as grooved micropatterns, can induce directional cell migration.^21,22^

Surface topography plays also a crucial role in bacterial adhesion and biofilm formation, although the precise relationship between surface features and bacterial response—whether repelling, attracting, or killing bacteria—remains complex and contradictory.^9^ A general statement, however, is that micrometric features similar to or larger than the bacteria size (1–2 μm) tend to enhance bacterial attachment by maximizing the surface-bacteria contact area.^23^ Oppositely, features in the submicrometric or nanometric scale (smaller than bacterial dimensions) typically reduce the contact area and inhibit bacterial adhesion, creating antifouling surfaces.^24^ However, this simplified size-dependent model is not universal, as other factors, such as physicochemical forces, cell membrane deformation, wettability, and topography-induced ordering, simultaneously influence bacterial-surface interactions.^25^ Additionally, surfaces with high-aspect-ratio nanoscale features can induce a bactericidal effect by physically disrupting bacterial membranes (i.e., bactericidal surfaces).^26^ Nonetheless, note that this contact-killing mechanism strongly varies depending on each bacterial strain and material type.

In this respect, surface modification stands out as a relevant strategy for designing implants with improved biological outcomes. The most common techniques for topographical modification of 3Y-TZP include grinding, sandblasting, chemical etching, and laser patterning.^27^ Among these, femtosecond (fs) laser patterning, particularly direct laser interference patterning (DLIP), offers unique advantages.^28^ This technique enables the creation of precise, high-aspect-ratio structures with minimal material damage, producing reproducible patterns (e.g., grooves,^21^ grids,^29,30^ pits^31^) at high processing speed.^27^ As a matter of fact, several studies have demonstrated that laser-assisted patterning of zirconia proves useful for enhancing cell adhesion, spreading, and differentiation.^21,27,31–34^ On the contrary, studies investigating the antibacterial properties of laser-modified zirconia are scarcer, with only some recent reports available,^32,35^ although the existing studies on other relevant biomaterials suggest a promising potential to interfere with bacterial attachment with this technique too.^36–38^

Notwithstanding the potential of topographical patterning, it is also common that a surface that promotes eukaryotic cell adhesion may facilitate bacterial attachment. Thus, a balance between improving cell response and simultaneously preventing bacterial colonization is required; however, this is yet an unmet challenge, especially for zirconia-based biomaterials.

Hence, in this study, ultrashort-pulsed-direct laser interference patterning (USP-DLIP) was employed to create well-defined micropatterns on zirconia that both enhance cell response and provide antibacterial properties. Four different patterns were created, varying in structural types (lines and grids) and periodicity (3 and 10 μm). The selection of these microstructure periodicities was based on the work of Minguela et al.,^21^ which focused only on the effect of the linear patterns on cell response but did not assess bacterial adhesion. Additionally, we included grid-like structures to explore their effect on both cell and bacterial behavior. The influence of the topographical parameters (structure type, periodicity, depth, and nanoroughness) on human mesenchymal stem cell (hMSC) response (adhesion, morphology, and migration) and bacterial adhesion was evaluated. Finally, a coculture study of hMSCs with Pseudomonas aeruginosa (P. aeruginosa) was performed to simulate a clinical scenario of postimplantation infection.

## 2 Experimental Procedure

### 2.1 Material Processing

Commercially available zirconia powder stabilized with 3 mol % yttria (TZ-3YSB-E, Tosoh) was employed to produce 15 mm disc specimens. The samples were prepared by cold isostatic pressing at 300 MPa in a cylindrical mold and subsequently sintered at 1450 °C for 2 h (6 °C/min heating and cooling rates). Then, all the samples were ground and mirror-like polished with diamond suspensions of decreasing particle size (30–6–3 μm) and a final alumina (Al_2_O_3_) suspension step (0.02 μm particle size). Finally, the samples were cleaned in an ultrasonic bath with 4 min washes in different solvents, as follows: cyclohexane (×3), acetone (×3), deionized water (×3), ethanol (×3), and acetone (×3), and then dried with nitrogen (N_2_).

### 2.2 Laser Patterning

The laser patterning of the samples was carried out using an ultrashort pulsed laser working in direct laser interference patterning configuration (USP-DLIP) in an air atmosphere. The laser system employed was a Ti:sapphire laser source (*Solstice ACE* by *Spectra Physics*) emitting ultrashort pulses of 150 fs at full width at half-maximum (fwhm) with a centered wavelength (λ) of 800 nm. Two-beam laser interference was achieved by using the optical setup described by Müller et al.^28^ Briefly explained, the optical configuration is composed of: (1) an aperture that defines the beam diameter, (2) a plate that adjusts the laser beam polarization perpendicular to the final pattern orientation, (3) a diffractive optical element (DOE) that divides the beam into two coherent beams, and (4) a lens system that directs and overlaps the two individual coherent beams on the sample surface. The overlapping of the two beams produces line-like interference patterns, whose periodicity (Π) is defined by eq 1.

1 $$\Pi =\frac{\lambda }{2\;\tan\;\left(\frac{\theta }{2}\right)}$$

where θ is the incidence angle between the individual beams.^39^ Therefore, the periodicity (Π) can be adjusted by changing the distance between the DOE and the focus lens system.

Four different samples were produced by varying the periodicity (3 and 10 μm) and the pattern shape (lines and grids). To do so, the optical setup and laser parameters (laser power, scan speed, and pulse overlapping) were adjusted to obtain homogeneous structures of the desired periodicity. First, two linear patterns of 3 and 10 μm periodicity were produced, which are labeled as L3 and L10, respectively. Then, two additional 3 (G3) and 10 μm (G10) periodic grid patterns were produced by rotating the L3 and L10 linear patterns 90° (respectively) and readjusting the laser parameters until homogeneous grids were obtained. A detailed summary of the samples and the laser parameters employed in each condition is included in Table 1.

**Table 1:** Summary of the Sample Codes and the Laser Parameters Employed during USP-DLIP Processing^a^

**sample**	**description**		**laser power** (mW)	**repetition rate** (kHz)	**overlap** (μm)	**scan speed** (mm/s)
**L3**	3 μm periodic lines		76	1	39	8
**G3**	3 μm periodic grids	1st	76	1	39	8
		2nd	44	1	40	18
**L10**	10 μm periodic lines		830	2.5	49	10
**G10**	10 μm periodic grids	1st	830	2.5	49	10
		2nd	630	2.5	49	20
**CTRL**	*mirror-like polished 3Y-TZP					

a For the grid patterns, 1st corresponds to the first laser scan, and 2nd to the second laser scan after rotating the samples 90°.

### 2.3 Postprocessing Storage and Cleaning

To ensure the stability of the surface properties and minimize potential contamination after laser processing, additional postprocessing steps were followed. Immediately after laser treatment, and just before physicochemical and biological characterization, the samples were cleaned in an ultrasonic bath with 3 min washes in different solvents (cyclohexane (×3), acetone (×3), deionized water (×3), ethanol (×3), and acetone (×3)), followed by drying with nitrogen (N_2_). After the cleaning, the samples were stored in a sealed container protected from ambient contamination.

### 2.4 Physicochemical Characterization

The topography of the patterns was visualized by field emission scanning electron microscopy (FESEM, Carl Zeiss Neon 40) to characterize the morphology of the linear and grid patterns and the laser-induced damage. Subsurface damage was also characterized by cross-sectional analysis. A site-specific cross-section was prepared using focused ion beam (FIB, FEI Helios Nanolab Dualbeam 600) milling and subsequently examined by FESEM. Furthermore, the topographical properties and roughness of the different USP-DLIP patterns were analyzed by confocal laser scanning microscopy (CLSM, Olympus Lext Ols41000). The CLSM measurements were performed using the 50× magnification lens and altering the digital post magnification between 2× and 6× at a laser wavelength of 405 nm. Three specimens of each condition were used, and 3 images per sample were taken for statistical significance.

The wettability of the surfaces was determined by static contact angle (SCA20, Dataphysics) and Milli-Q water was used as a wetting liquid at a fixed droplet volume of 1 μL. On the linear patterns (L3 and L10), water contact angle (CA) was measured following the parallel (θ_=_) and perpendicular (θ_⊥_) directions of the grooves, while on the grid patterns (G3 and G10), CA was measured along the two orientations of the crossing grooves (0° (θ_=_) and 90° (θ_⊥_)). For each condition and groove direction, 9 drops were measured (3 samples per condition ×3 measures per sample).

Since the patterned samples (L3, G3, L10, G10) were not smooth, and a Wenzel model was applied to calculate the intrinsic contact angle (θ), using eq 2.

2 $$\cos\theta_{m}=r\;\cos\;\theta$$

where θ_m_ is the measured contact angle and *r* is the roughness factor.

The roughness factor is defined as the relationship between the real surface area/projected surface area, and it is calculated using eq 3.

3 $$r=1+\frac{S_{\mathrm{dr}}}{100}$$

where *S*_dr_ is developed surface area.

Finally, the difference between the parallel and the perpendicular contact angles was calculated as (Δθ = |θ_⊥_–θ_=_|) as a parameter to quantify the surface anisotropy.

X-ray photoelectron spectroscopy (XPS) was used to determine the elemental composition of the surface after laser irradiation and to evaluate potential alumina contamination from the polishing process. The detection of C, O, Zr, Y, and Al elements was conducted using an XPS system (SPECS Surface Nano Analysis system GmbH, Berlin, Germany) equipped with a nonmonochromatic Mg anode ×50 source, operating at 150W and a Phoibos 150 MCD-9 detector. Spectra were recorded with pass energy of 25, 0.1 eV steps and a pressure below 7.5 × 10^–9^ mbar. Casa XPS software was used to analyze the data and calculate the atomic percentages. Prior to analysis, C 1s spectra were calibrated at 284.4 eV and the rest of the peaks were referenced to such an energy.

### 2.5 Biological Characterization

#### 2.5.1 Cell Culture

Bone-marrow-derived human mesenchymal stem cells (hMSCs) (Zen-Bio, HBMMSC-F) were cultured in advanced Dulbecco’s modified Eagle’s medium (DMEM) supplemented with 10% (v/v) fetal bovine serum (FBS), 1% (w/v) penicillin/streptomycin and 1% (w/v) l-glutamine. Cells were cultured at 37 °C in a humidified atmosphere containing 5% (v/v) CO_2_ and the culture medium was changed 3 times per week. After reaching a confluence of 90%, the cells were detached using trypLE-EDTA.

Before the cellular experiments were conducted, the samples were sterilized in 70% (v/v) ethanol for 15 min, washed three times with phosphate-buffered saline (PBS), and transferred to a sterile 24-well plate to carry out the experiments.

#### 2.5.2 Cell Adhesion

hMSCs at passage 4 were seeded at a concentration of 5.000 cells/well in a serum-free medium and incubated at 37 °C and 5% (v/v) CO_2_-containing atmosphere. After 6 h of incubation, cells were rinsed with PBS and fixed with paraformaldehyde (PFA, 4% w/v in PBS) for 30 min.

Immunofluorescence staining was performed to assess cell adhesion and morphology. Cell were first permeabilized with 0.05% (w/v) Triton X-100 in PBS for 20 min followed by a 1 h blocking step with bovine serum albumin (BSA, 1% (w/v) in PBS). The cytoskeletal actin filaments (F-actin) were stained with phalloidin–Alexa Fluor 546 (1:300, in triton 0.05%, 1 h), focal adhesions (FAs) were stained with mouse antivinculin (1:400, in BSA 1%, 1 h) followed by Alexa 488 goat antimouse IgG antibody (2 drops/mL, in triton 0.05%, 1 h), and the nuclei were stained with 4′,6-diamidino-2- phenylindole (DAPI) (1:1000, in PBS-glycine 20 mM) for 2 min. The staining steps were done in the dark, and the samples were washed with PBS-glycine between all the steps (3 times per 5 min each). Finally, the samples were mounted in Mowiol and examined by a fluorescence LSCM (Carl Zeiss LSM 800). The 5× objective was employed to characterize the cell adhesion (i.e., cell numbers), 10× magnification was used to examine cell area (i.e., cell spreading) and morphology, and 40× magnification was used to visualize FAs.

The experiment was carried out in triplicates. Five images per sample were taken to analyze the adhesion (5×) and morphology (10×), and the Fiji/ImageJ image processing package was used to quantify cell adhesion and obtain cell-shape parameters.

Once all images were obtained, the cells were dehydrated through immersion in increasing concentrations of ethanol, coated with a 15 nm layer of gold, and visualized by FESEM.

#### 2.5.3 Cell Migration

hMSCs migration assay was conducted to evaluate the migration and wound healing capacity of the cells when cultured on different surfaces. A two-well containing silicon culture insert (Ibidi) was placed on the surface of the samples. 10.000 hMSCs at passage 5 were seeded in the complete medium in each well of the inset and cultured for 24 h to attach to the surface. After this time, the insets were removed from the samples, leaving two confluent squares separated by a gap of 500 μm. At this point, one sample was carefully rinsed with PBS to remove the nonadherent cells, fixed with PFA (4% w/v in PBS, 30 min), and stored at 4 °C in PBS. This is the reference sample that is used to verify the size and shape of the initial layer just after removing the inset. Then, the rest of the specimens were cultured for another 72 h in complete medium to allow the cells to start migrating along the surface. Then, the samples were rinsed carefully with PBS, fixed with PFA (4% w/v in PBS, 30 min), and stored at 4 °C in PBS.

Cells were permeabilized with 0.05% (w/v) Triton X-100 in PBS for 20 min and stained in a 0.1% (w/v) crystal violet solution for 10 min. The samples were rinsed (>10 times) until the excess dye was removed and dried in a N_2_ stream. Cell migration was characterized in terms of wound healing capability (wound closure) and migration length (along the *x* and *y*-axis). The wound closure and migration length were visualized under a stereomicroscope system (Olympus SZX16) and analyzed with Fiji/Image-J package.

#### 2.5.4 Bacterial Culture

Gram-positive Staphylococcus aureus (S. aureus) and Gram-negative P. aeruginosa were selected as model microorganisms for bacteria adhesion assays. S. aureus and P. aeruginosa were grown overnight at 37 °C in Brain Heart Infusion Broth (BHI, Difco) and Luria Broth (LB, Scharlab), respectively, prior to each assay.

Before conducting the experiment, the samples were sterilized by immersing them in 70% ethanol (v/v) for 15 min, washed three times with PBS and transferred into a sterile 12-well plate.

#### 2.5.5 Bacterial Adhesion

The antibacterial properties of the USP-DLIP samples were evaluated by a live/dead staining assay. The optical density at 600 nm (OD600) was adjusted to 0.2 ± 0.1, corresponding to a bacterial concentration of 10^8^ colony-forming units per mL (CFU/mL). Next, the bacteria were diluted to the respective seeding concentrations (S. aureus 10^6^ CFU/mL; P. aeruginosa 10^6^ CFU/mL), seeded on the samples (1 mL/well), and incubated during 4 h at 37 °C. After the incubation time, the samples were washed thrice with PBS to remove nonadherent bacteria. Then, the adhered bacteria were fixed with glutaraldehyde (2.5% w/v in PBS) for 20 min, washed three times with PBS and stored at 4 °C in PBS.

Bacteria viability was assessed by using the LIVE/DEAD BackLight Bacterial Viability Kit (ThermoFisher). The kit contains two dyes: (1) propidium iodide, a red-fluorescent nucleic acid dye that only penetrates damaged membranes, and (2) SYTO9, a green-fluorescent nucleic acid dye that stains all bacteria, as it can penetrate both intact and damaged membranes. For the staining procedure, the bacteria were incubated in a solution containing propidium iodide and SYTO9 (3 μL of kit/mL PBS) at room temperature for 15 min in the dark. Subsequently, the samples were washed three times with PBS and bacterial viability was examined by fluorescence LSCM (Carl Zeiss LSM 800) using 63× magnification.

The experiments were carried out in triplicates, and five images per sample were taken for the analysis. Fiji/ImageJ was used to quantify the bacteria’s attachment to the different surfaces. The total area covered by bacteria was calculated (% area of green and red fluorescence) on each sample and compared to the one of polished 3Y-TZP (CTRL).

After the live/dead analysis, the bacteria were dehydrated through immersion in increasing concentrations of ethanol, coated with a 15 nm layer of gold, and visualized by FESEM.

#### 2.5.6 Cell–Bacteria Coculture

Bacteria and hMSCs were cocultured following the *infection postimplantation* coculture method described by Piñera-Avellaneda et al.^40^ hMSCs (Zen-Bio, HBMMSC-F) at passage 4 were cultured in Advanced DMEM supplemented with 10% (v/v) FBS, 1% (w/v) penicillin/streptomycin and 1% (w/v) l-glutamine. P. aeruginosa bacteria were selected for the coculture assay. Bacteria were grown aerobically overnight in antibiotic-free Advanced DMEM (+1% FBS, + 1% Glu, no P/S) at 37 °C in a shaker incubator at 220 rpm (RPM). Then, the bacterial suspension was diluted in the same medium to OD600 = 0.1 and incubated to reach the midexponential phase (approximately 10^8^ CFU/mL). The sample sterilization procedure was performed as previously explained.

First, hMSCs were seeded on the surfaces at a concentration of 15.000 cells/well and incubated for 24 h at 37 °C in complete Advanced DMEM (+10% FBS, + 1% Glu, + 1% P/S). After incubation, the cell medium was removed, and samples were rinsed thrice with PBS. Then, the bacteria were diluted to a concentration of 10^3^ CFU/ml, seeded on the samples (1 mL/well) in antibiotic-free DMEM (+1% FBS, + 1% Glu, no P/S), and incubated for 2 h at 37 °C and 5% CO_2_. After this time, the samples were rinsed with PBS and fixed with PFA (4% w/v in PBS) for 30 min.

Immunofluorescence staining was performed to assess cell adhesion and morphology of the cells in coculture. Fluorescence staining of cell nuclei and actin filaments was carried out as indicated in Section 2.5.2. The samples were examined by fluorescence LSCM (Carl Zeiss LSM 800), using 5× (cell number) and 10× (cell area) objectives. Finally, the images were analyzed with Fiji/ImageJ.

### 2.6 Statistical Analysis

Minitab software was used for statistical analysis, in which the statistical differences (*p* < 0.05) among the conditions of the study were assessed by ANOVA using the Tukey test.

## 3 Results

### 3.1 Surface Characterization

#### 3.1.1 Topographical Characterization

Four distinct patterns were created on 3Y-TZP via femtosecond laser DLIP modification, with variations in surface structure and periodicity, to evaluate the influence of these topographical features on the cellular and bacterial responses. In detail, the generated laser structures include two linear patterns (L3 and L10) and two grid patterns (G3 and G10), with periodicities of 3 and 10 μm, respectively. Polished 3Y-TZP was used as the control (CTRL).

The resulting surface topographies are illustrated in Figure 1a. In all cases, the laser-patterned surfaces exhibited well-defined, reproducible, and periodically spaced microstructures. For the linear ones (L3 and L10), the surface consisted of periodically alternating peaks (unaffected by the laser) and valleys (ablated areas). In contrast, both grid patterns (G3 and G10) display square-shaped unaltered regions separated by laser-ablated cross-section valleys.

**Figure 1:**
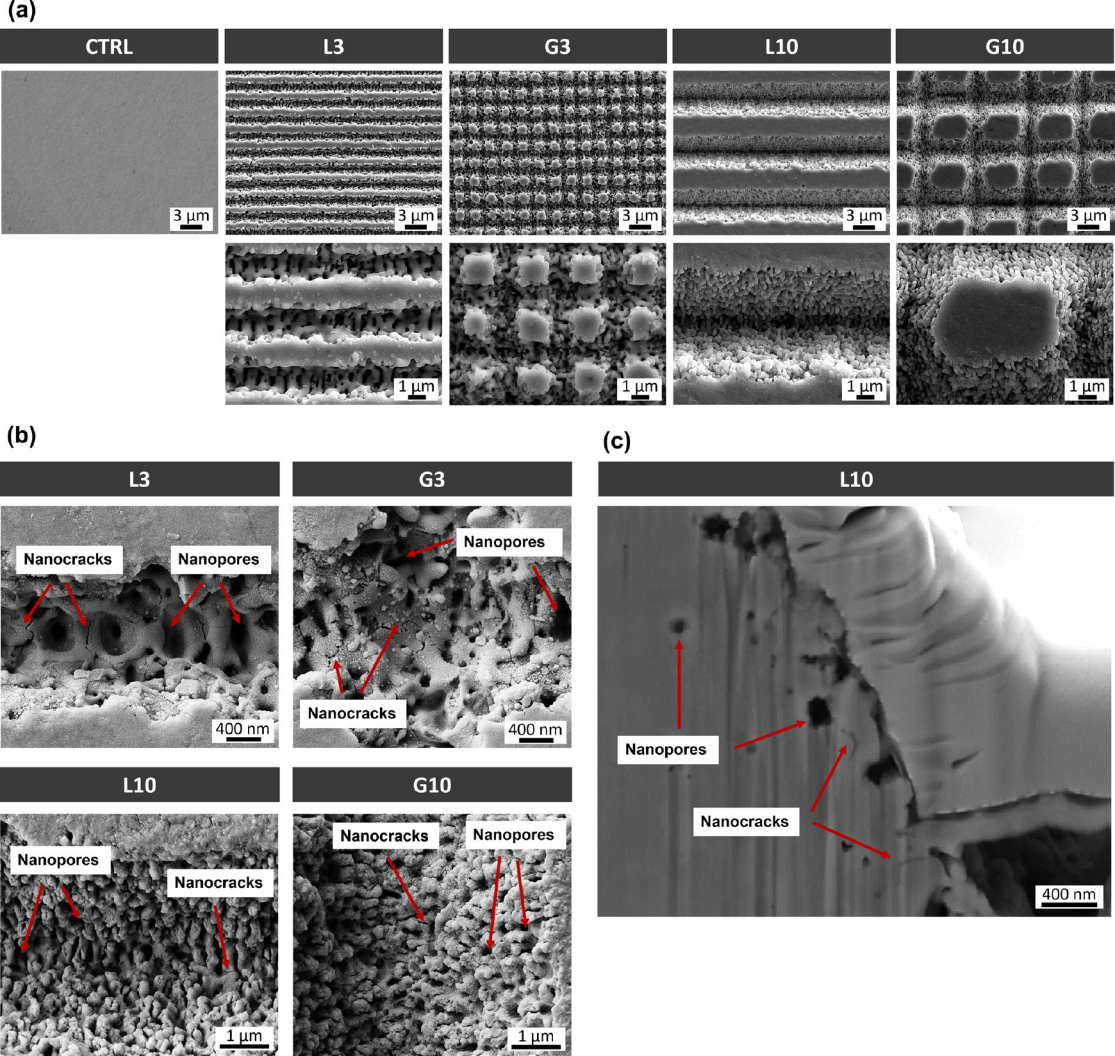
(a) FESEM micrographs of the polished surface (CTRL) and linear (L3 and L10) and grid (G3 and G10) patterns generated by the USP-DLIP technique. (b) FESEM images of the pattern valleys indicating surface damage induced during the laser processing. (c) FESEM micrograph of the cross-section of sample L10 showing the subsurface damage induced in the laser grooves.

The most relevant topographical characteristics are summarized in Table 2, including average surface roughness (*S*_a_), periodicity, valley depth and width, and valley roughness (*R*_q_) (see Supporting Information S1). Additionally, topography images and profiles of the micropatterns obtained by CLSM are provided in Supporting Information S2, further illustrating the structural characteristics of the laser-modified patterns. The measured periodicities of the micropatterns are very close to those of the target ones. For the linear patterns, periodicities of 3.04 (L3) and 9.81 μm (L10) were measured. In the grid patterns, the periodicity was calculated along the two structural directions, showing no significant differences along the axes. In particular, the periodicities of G3 were 2.98 and 2.88 μm in each direction, while for G10, they were 9.84 and 9.81 μm.

**Table 2:** Results from the Topographical Analysis Performed by CLSM^a^

	**CTRL**	**L3**	**G3^b^**	**L10**	**G10^b^**
***S*_a_ (μm)**	0.01 ± 0.00	0.36 ± 0.02	0.29 ± 0.02	1.10 ± 0.05	1.05 ± 0.07
**periodicity (μm)**		3.04 ± 0.02	2.98 ± 0.07	9.81 ± 0.10	9.84 ± 0.04
			2.88 ± 0.18		9.81 ± 0.04
**depth (μm)**		1.20 ± 0.09	1.07 ± 0.06	3.16 ± 0.07	3.15 ± 0.14
			0.95 ± 0.06		2.19 ± 0.18
**width (μm)**		1.45 ± 0.02	1.49 ± 0.08	5.09 ± 0.19	5.25 ± 0.23
			1.54 ± 0.06		5.10 ± 0.34
***R*_q_ valley (nm)**		206 ± 6	160 ± 11	315 ± 41	284 ± 18
			162 ± 7		256 ± 15

a In the table, *S*_a_ and *R*_q_ indicate the average surface roughness and root mean square of the heights, respectively. A schematic of the topographical parameters characterized in the linear and grid patterns is included in supplementary material S1.

b In the grid patterns, groove periodicity, depth, and *R*_q_ were measured along the grooves generated during the first scan (first row) and the cross-section grooves generated during the second scan (after rotating the sample 90°) (second row).

Both the valley depth and width remained consistent within structures of the same periodicity but varied across patterns with different periodicities (Table 2). In L3 and G3, the valley depth and width were approximately 1 and 1.5 μm (respectively), while for L10 and G10 samples, the measured depth and width were approximately 3 and 5 μm (respectively). Furthermore, the valley depth showed slight variation in the grid patterns between the two groove directions, while the width remained consistent. This variation was more pronounced in G10, where a slight reduction in depth occurred in one groove direction. In concrete, these shallower valleys were formed during the second laser scan (after the sample was rotated 90°), since the laser power was reduced to decrease undesired surface deterioration by incubation effects from the previous processing step.

Overall, the four laser-treated surfaces displayed a higher surface roughness (*S*_a_) compared to polished 3Y-TZP. Furthermore, the *S*_a_ increased according to pattern size. 3 μm periodic patterns (L3 and G3) exhibited a *S*_a_ around 0.3 μm, while those with 10 μm periodicity (L10 and G10) showed significantly higher roughness, around 1 μm.

In addition to the microscale structures, nanoscale topography was also generated within the valleys due to the laser ablation process. As depicted in Figure 1b, the morphology of the laser-induced nanotopography differs among the laser-modified specimens. L3 pattern exhibited periodic line-like nanostructures inside the valleys, known as nanoripples, of a periodicity of about 500 nm (see Figure 1b, L3) and with a corresponding ablation-induced roughness (*R*_q_) of 206 nm (see Table 2). In the case of the 3 μm grid pattern (G3), such a nanoripple-like structure was maintained between the grids, but it was annihilated in the cross-section. As a result, the valley roughness (*R*_q_) decreased to 160 nm (Table 2). In contrast, the nanotopography on the valleys of L10 and G10 patterns consisted of many nanocavities, with corresponding *R*_q_ values of 315 and 284 nm, respectively.

Furthermore, the high-magnification SEM images evidenced the presence of surface damage induced by laser processing. As indicated in Figure 1b, nanosized cracks and cavities appeared along the laser valleys in all the linear and grid patterns. When comparing these patterns, L3 and G3 exhibited a higher density of nanocracks, whereas L10 and G10 showed a significantly greater density of nanopores with minimal presence of nanocracks. Additionally, the laser-induced subsurface damage was visualized via FIB cross-sectioning. Figure 1c revealed the presence of nanopores and nanocracks below the surface confined within the first 1 μm layer.

#### 3.1.2 Surface Chemistry

Chemical composition analysis was performed to evaluate potential changes in the material’s surface after laser irradiation. The detected elements with their atomic % are summarized in Figure 2a. The results showed a significant increase in carbon (C) content after laser treatment, while oxygen (O) and zirconium (Zr) contents slightly decreased. Correspondingly, the yttria (Y) content showed a slight decrease, maintaining the characteristic Y/Zr ratio of the 3Y-TZP ceramic. Furthermore, the presence of aluminium (Al) was very low (between 0.0 and 0.3 atomic %), indicating minimal contamination from the polishing step with alumina suspension.

**Figure 2:**
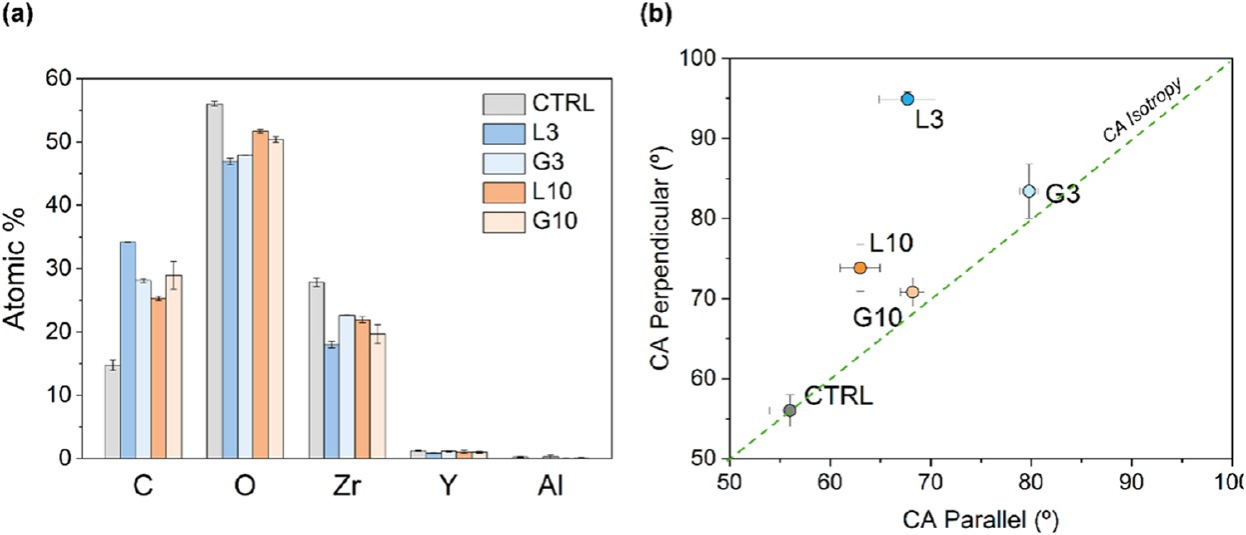
(a) Analysis of the chemical composition (atomic percentages) by XPS. (b) Intrinsic water contact angles of the samples. In the case of the micropatterns (L3, G3, L10, and G10), CA was measured in the parallel and perpendicular direction of the grooves.

#### 3.1.3 Wettability

The sessile drop method was used to measure the static contact angle (CA) of patterned 3Y-TZP at two orientations, parallel and perpendicular. Then, the Wenzel equation was used to calculate the intrinsic CA of the different surfaces (Figure 2b). A detailed summary of the wettability is presented in Table S3.1 (Supporting Information), which includes the measured CA (both directions), the intrinsic CA calculated using the Wenzel equation (in both directions), and the surface wettability anisotropy (Δθ).

As observed in Figure 2b and Table S3.1, the CTRL surface had a CA of 55.6 ± 2.2°, while all the patterned surfaces presented a more hydrophobic behavior with CA ranging from 63.0° up to 94.60°. Among the patterns, the low periodicity structures (L3, G3) were slightly more hydrophobic than the high periodicity ones (L10, G10).

Unlike the grid patterns (G3, G10), which showed similar CA values in both measured directions, the linear patterns (L3 and L10) induced anisotropy in the wetting behavior. In these samples, the CA was higher in the perpendicular direction than in the parallel one. The water droplets adopted an elongated shape, spreading more easily along the grooves (parallel direction) while being pinned by the peaks of the pattern. Additionally, this anisotropic behavior was more pronounced in L3 (Δθ = 27.2) than in L10 (Δθ = 10.8) (see Supporting Information S3). While both exhibited similar CA in the parallel direction, the CA in the perpendicular direction was significantly higher for L3 (94.9°) compared with L10 (73.8°), resulting in higher anisotropy.

### 3.2 Cell Response

#### 3.2.1 Cell Adhesion

A cell adhesion assay was conducted to evaluate the impact of the patterns on hMSCs attachment, spreading, and morphology. A clear enhancement of hMSCs adhesion was observed on the linear (L3 and L10) and grid (G3 and G10) surfaces when compared to the polished one (CTRL) (Figure 3a). In particular, cell attachment was increased 1.5-fold on all the laser-structured samples, but no significant differences were observed among the different topographies (*p* < 0.05). Similarly, the cells seeded on the patterned surfaces exhibited enhanced spreading compared to the polished surface (Figure 3d). The cells on the micropatterns exhibited a significantly larger cell body area, which was increased by a factor of 1.5 (G3, L10, and G10) and 2 (L3), according to each topography (Figure 3b). Despite the 3 μm periodic pattern showing the best results in terms of cell spreading (i.e., cell area), the differences were not statistically significant (*p* < 0.05) when compared to the other linear and grid structures.

**Figure 3:**
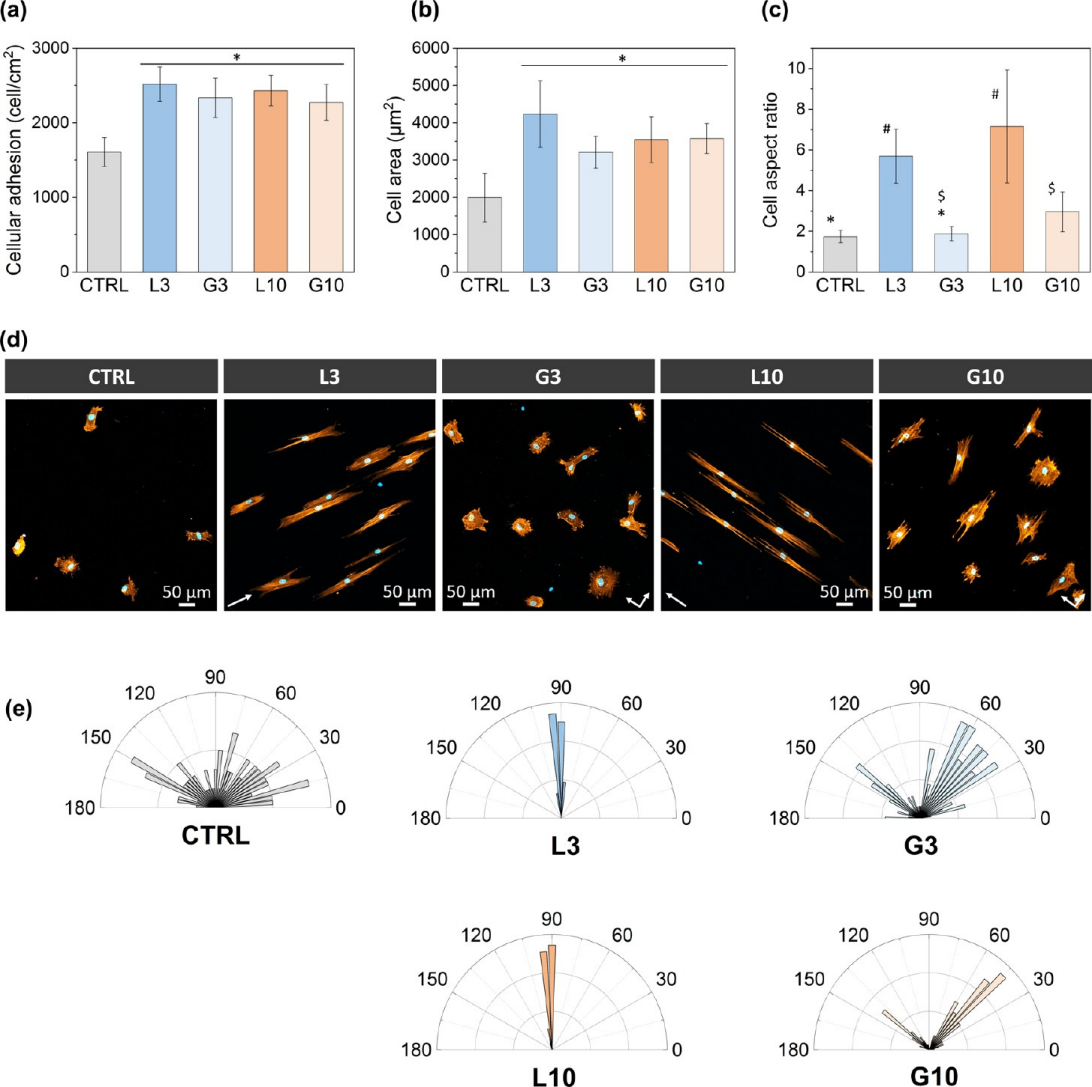
hMSC adhesion results after 6 h of incubation in a serum-free medium. (a) Cellular adhesion (cell/cm^2^), (b) cell area (μm^2^), and (c) cell aspect ratio. In the graphs, groups sharing the same symbol (*, # or $) show no significant differences (*p* < 0.05). (d) Representative fluorescence images of the adhesion test, with cell nuclei stained with DAPI (blue) and actin filaments with phalloidin–Alexa Fluor 546 (orange). White arrows indicate groove and grid directions. (e) Angular histogram of cell orientation showing the relative number of cells oriented at specific angles. For linear patterns (L3 and L10), 90° corresponds to the groove direction. For grid patterns (G3 and G10) the crossing grooves correspond to 45 and 135° orientations.

The morphology and alignment of the cells were dependent on the pattern structure type, as evidenced by the fluorescence images (Figure 3d) and SEM micrographs (Figure 4a–d). On polished zirconia (CTRL), hMSCs exhibited a poorly developed cytoskeleton and rounded shape, with no preferential alignment due to the lack of topographical cues. In contrast, on the linear patterns (L3 and L10), the cells stretched along the grooves, leading to a significant increase in the cell aspect ratio (Figure 3c). Furthermore, these linear patterns induced strong cell alignment along the laser valleys. In fact, all cells on linear patterns perfectly aligned parallel to the grooves (Figure 3e). For the grid patterns (G3 and G10), the cells did not exhibit such an elongated morphology but instead spread across the entire surface. However, small differences were observed in the cell morphology between the two grid patterns. On G3, the cells displayed a well-spread but more rounded cell body, accompanied by multiple and small-size cytoskeletal extensions that extended in all directions. In contrast, on G10 the cells adapted more closely to the grid structure as they displayed larger cytoskeletal extensions growing in both grid directions. This resulted in a slight increase in the aspect ratio compared to G3 and CTRL, although not as pronounced as observed on the linear patterns (Figure 3c). Despite not having an elongated morphology, the grid patterns still influenced cell alignment. As shown in Figure 3e, on G3 and G10 samples, the cells were preferentially oriented along both directions of the grid structure (in the graph this corresponds with 45 and 135°). Interestingly, on both samples the alignment was more pronounced in one of these directions, which corresponds to the one containing the deeper valleys produced by the first laser scan.

**Figure 4:**
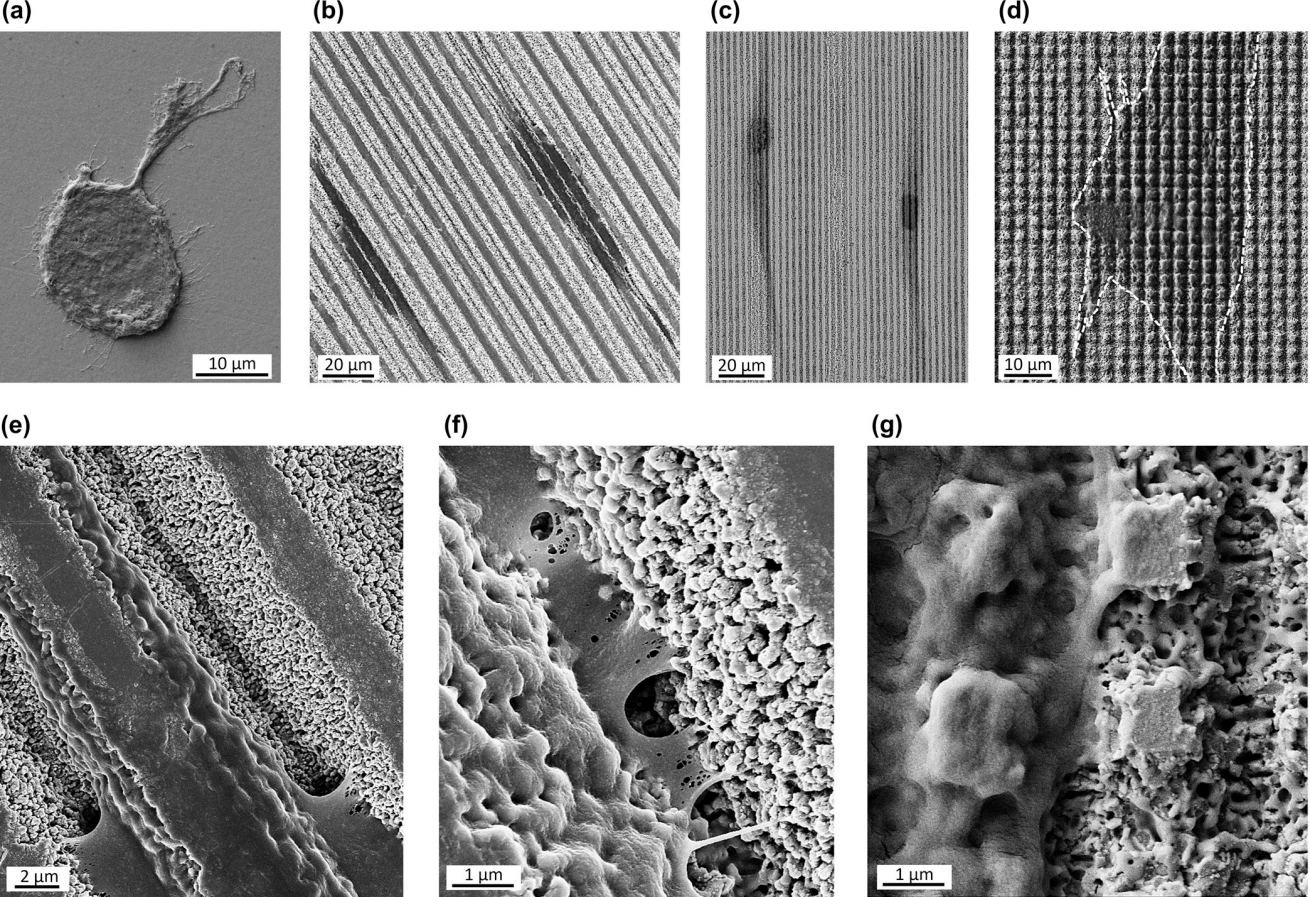
FESEM micrograph from the hMSCs adhesion test assay. (a) CTRL, (b) L10, (c) L3, and (d) G3. (e and f) Magnification of the cell adhered to L10 showing (e) the filopodia extending along the grooves and (f) the cell anchoring to the nanotopography. (g) Magnification of a cell adhered to G3.

The formation of focal adhesions (FAs) was assessed through vinculin staining to further investigate the cell-surface interaction in response to the different topographies. The cells adhering to the linear and grid patterns but not to the control presented nascent FA complexes (Figure 5). These protein complexes (i.e., vinculin) were mainly distributed at the periphery and tip of the cytoskeletal elongations. Also, on L10 and G10 samples, vinculin distribution followed the pattern shape and located at the nanocavities from the grooves rather than on the polished areas.

**Figure 5:**
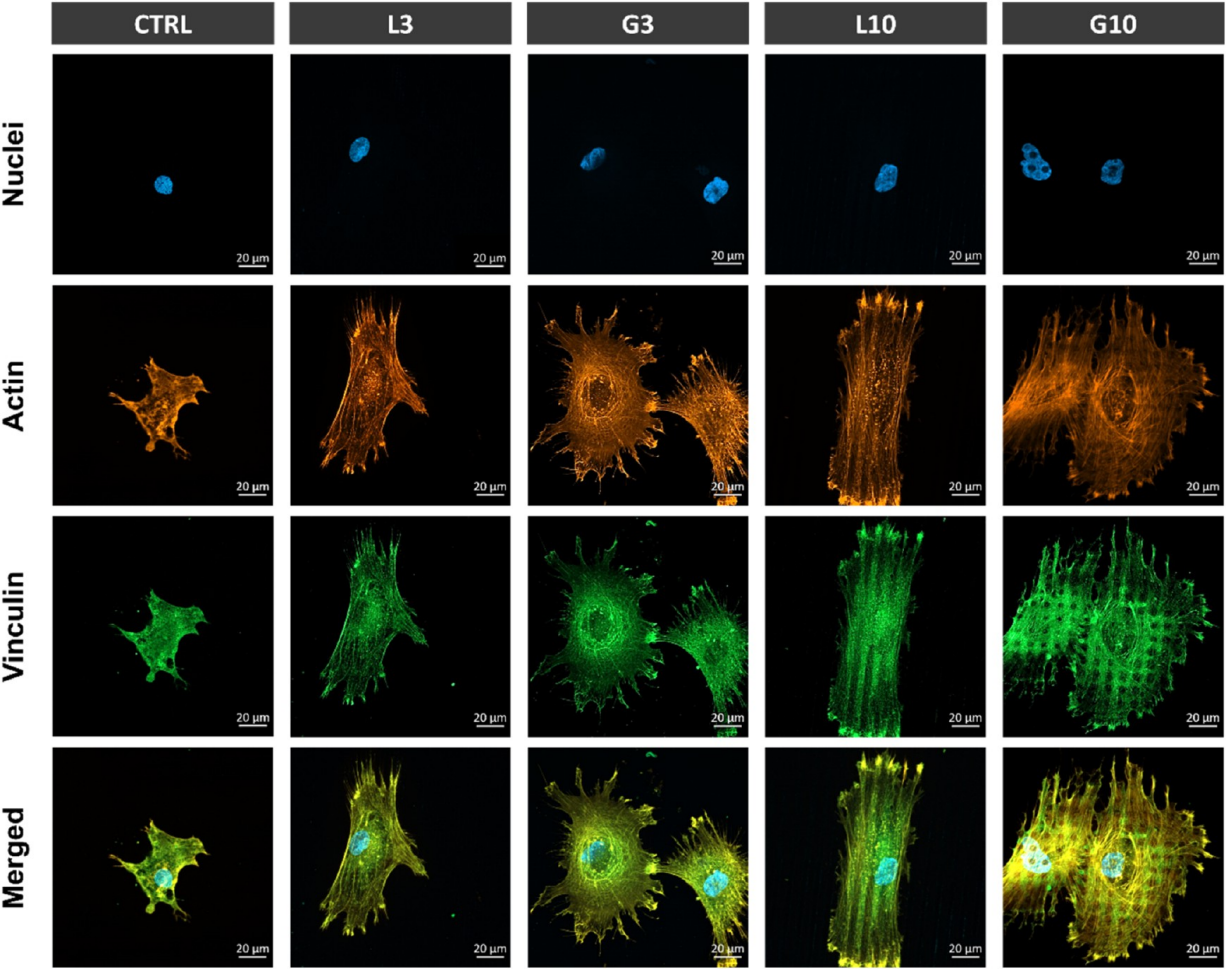
Immunostaining of focal adhesions (FAs) and morphology of hMSCs cultured for 6 h in serum-free medium on polished (CTRL) and laser-patterned linear (L3 and L10) and grid (G3 and G10) surfaces. In the fluorescence images, the nuclei of the cells were stained with DAPI (blue), actin filaments with phalloidin–Alexa Fluor 546 (orange), and FAs (vinculin protein) with Alexa Fluor 488 (green).

Additionally, high-magnification SEM images were obtained to better understand how the cells anchored to the different topographies. The nanotopography from the valleys played a pivotal role in the cell-surface adhesion process. As shown in Figure 4e,f, on the L10 linear pattern, filopodia extensions from the cells are anchored to the nanocavities within the laser valleys. Similarly, on the grid pattern (G3), the cells established contacts with the nanoripples located in the laser valley (Figure 4g).

#### 3.2.2 Cell Migration

hMSCs migration was evaluated to assess the migration capacity of the cells cultured on different surfaces. A two-well containing silicon insert was used for the assay, and hMSCs migration was evaluated in terms of migration length (along the *x* and *y* axes) and wound closure (the wound between the wells) (Figure 6a).

**Figure 6:**
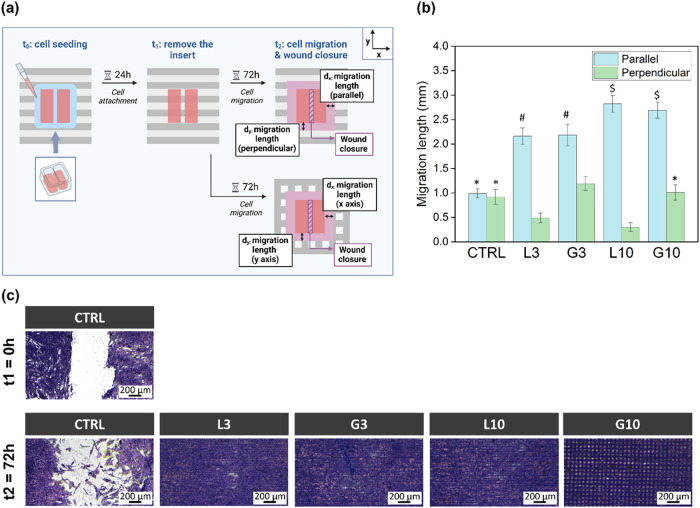
Results from the cell migration assay. (a) Schematic of the cell migration assay procedure. (b) hMSC migration length (mm) along the parallel (*x*-axis) and perpendicular (*y*-axis) directions (see schematics from (a)). (c) Optical images from the wound healing results (gap closure). The cells were stained with crystal violet (purple). CTRL *t*_1_ = 0 h shows the reference cell gap that is obtained just after removing the inset (the same gap was obtained for the rest of the conditions, not shown). The images from the second row indicate the wound closure achieved after cell migration for 72 h.

As illustrated in Figure 6b, the linear and grid patterns promoted anisotropic migration, whereas cells on the polished surface exhibited isotropic and reduced migration. Additionally, migration behavior differs according to the type of laser structure. Both linear patterns showed the highest anisotropy, particularly L10, where cell migration was significantly enhanced in the direction parallel to the laser valleys (*d*_*x*_), but was strongly hindered across to the grooves (*d*_*y*_) (i.e., perpendicularly). Specifically, in the direction parallel to the grooves, the migration length was doubled (L3) and nearly tripled (L10) compared to the polished sample (CTRL), while the displacement in the perpendicular direction was reduced by half (L3) and third (L10). In the grid patterns, the grooves along the *x*-axis direction correspond to those generated in the first step (deeper grooves), while the grooves oriented along the *y*-axis were formed in the second step, after rotating the sample 90° (shallower grooves). Despite the presence of grooves in both directions, hMSCs still exhibited a noticeable anisotropic migration. Migration was thus enhanced along the deeper grooves (*d*_*x*_), showing a 2-fold increase for G3 and a 3-fold increase for G10, similar to the linear patterns. In contrast, along the *y*-axis (*d*_*y*_), cell displacement on G10 remained similar to that of CTRL and even slightly increased on G3.

Furthermore, the wound healing capability on different surfaces was also evaluated. As illustrated in Figure 6c, a discernible cell gap between the two wells was evident immediately after the removal of the inset (*t*_1_ = 0 h) for all conditions (data only shown for CTRL). Following a 72-h cultivation period (*t*_2_ = 72 h), a reduced number of cells on the polished surface (CTRL) started migrating, yet the gap remained clearly visible. On the contrary, complete wound closure was achieved on all of the patterns after 72 h, indicating a significant enhancement of hMSCs migration driven by the laser-induced topographies.

### 3.3 Bacterial Response

The bacterial response to the laser-patterned 3Y-TZP surfaces was evaluated through live/dead staining. The Gram-positive S. aureus and the Gram-negative P. aeruginosa bacteria were selected as representative models for the purpose of this study, as they are characteristic strains commonly associated with biomaterial-related infections.

Representative fluorescence images of S. aureus adhesion are shown in Figure 7a, where viable bacteria are stained in green and dead bacteria are stained in red. The images demonstrated a consistently low number of dead bacteria (red cells) in all the samples, with no remarkable differences between them. However, a notable reduction in the density of viable bacteria (green cells) was observed on the 3 μm periodic structures (L3 and G3) compared to polished zirconia (CTRL). In contrast, a decrease in bacterial colonization was not observed in the high periodicity patterns (L10 and G10). To further quantify this observation, the bacterial coverage area was measured and normalized to the value observed on the polished surface (CTRL). As shown in Table 3, the adhesion of S. aureus was reduced by around 30% on both low periodicity patterns (L3 and G3), but not on L10 and G10 samples, which exhibited similar levels of bacterial colonization to the polished surface.

**Table 3:** Percentage of Bacterial Adhesion Compared with Smooth 3Y-TZP (CTRL)

	**bacteria adhesion compared to CTRL (%)**				
	**CTRL**	**L3**	**G3**	**L10**	**G10**
S. aureus	100 ± 4	69 ± 9^a^	70 ± 7^a^	98 ± 5	94 ± 7
P. aeruginosa	100 ± 13	75 ± 6^a^	75 ± 13^a^	79 ± 13^a^	84 ± 3^a^

a Indicates statistically significant differences with respect to CTRL (*p* < 0.05).

**Figure 7:**
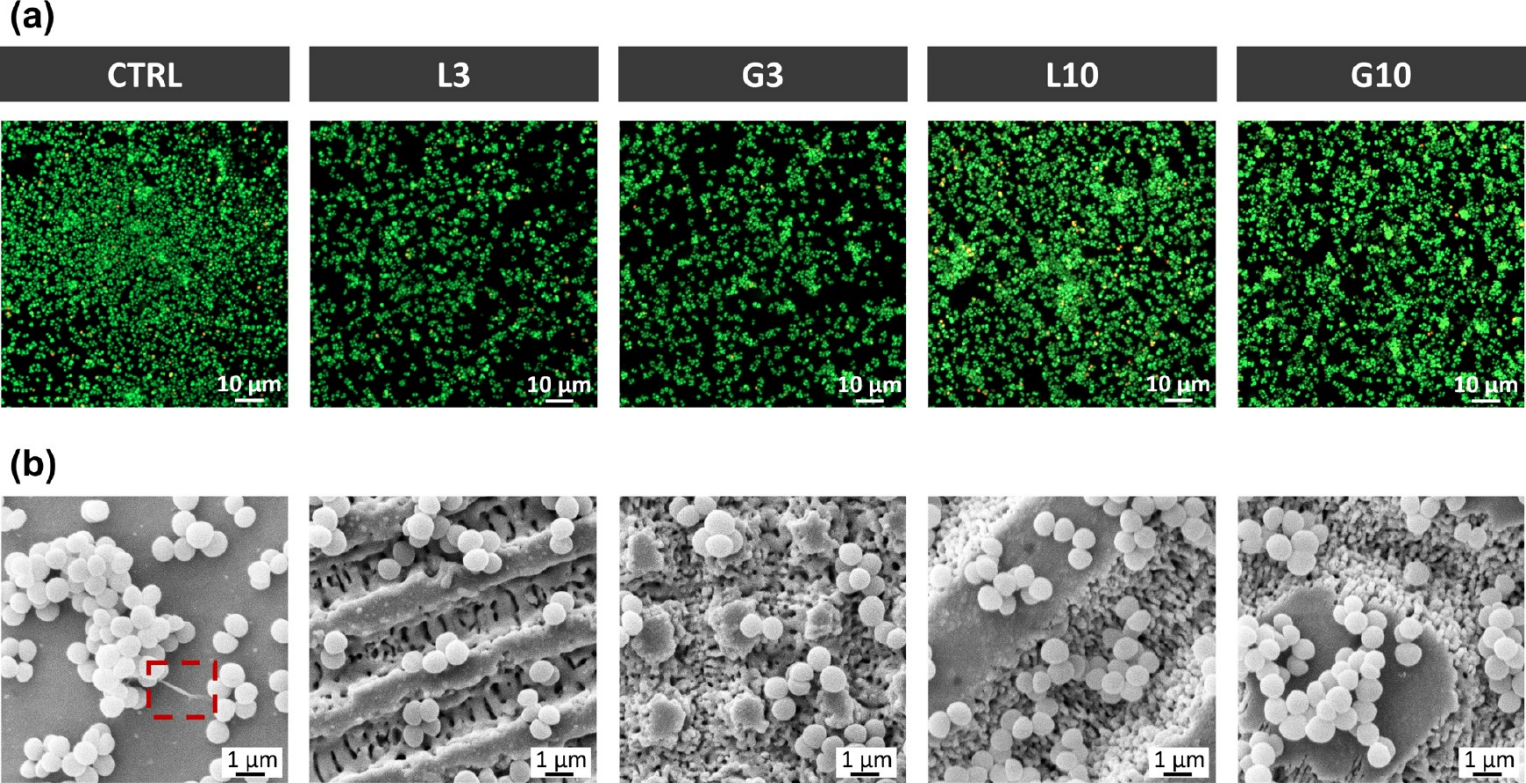
Results from the S. aureus adhesion assay. (a) Fluorescence CLSM images of the live/dead staining of S. aureus after 4 h of incubation on different surfaces. Live bacteria are stained with SYTO9 (green) and dead ones with propidium iodide (red). (b) SEM micrographs of S. aureus cultures on the different surfaces. Red square (CTRL) indicates the secretion of extracellular polymeric substance (EPS).

The effect of the patterns on the attachment and morphology of S. aureus was further investigated by SEM (Figure 7b). There was no evidence of bacterial damage in any of the samples, as the bacteria adhered to all the surfaces and presented the typical spherical morphology. Additionally, the microstructures of the patterns did not affect the bacterial distribution as bacteria were indistinctly distributed onto both the laser valleys and the unaltered top surface areas. Nevertheless, the laser-structured surfaces seemed to hinder S. aureus proliferation and aggregation in the low periodicity patterns. On the polished surface, the bacteria formed large 3D agglomerates and secreted extracellular polymeric substance (EPS), an indicator of effective binding to the surface (Figure 7b, red square). On L10 and G10, EPS secretion was not observed, but bacteria still were able to form large clusters. In contrast, L3 and G3 patterns apparently disrupted bacterial growth, as evidenced by the absence of large bacterial 3D clusters and EPS secretion.

Additionally, the antibacterial effect of the patterns against P. aeruginosa was also assessed. As shown in Table 3, the four micropatterns inhibited the early adhesion of P. aeruginosa in comparison to that on the polished surface. In particular, the greatest effect was achieved on L3 and G3, where the bacterial coverage area was reduced by 25%. Bacterial adhesion decreased to a lower extent on L10 and G10, with a reduction of 21 and 16%, respectively. The fluorescent images presented in Figure 8a demonstrated that the viability of the bacteria was not affected by the patterns, as evidenced by the minimal presence of dead cells across all surfaces. However, the overall bacterial density was significantly reduced on the micropatterns, particularly on the L3 and G3 surfaces.

**Figure 8:**
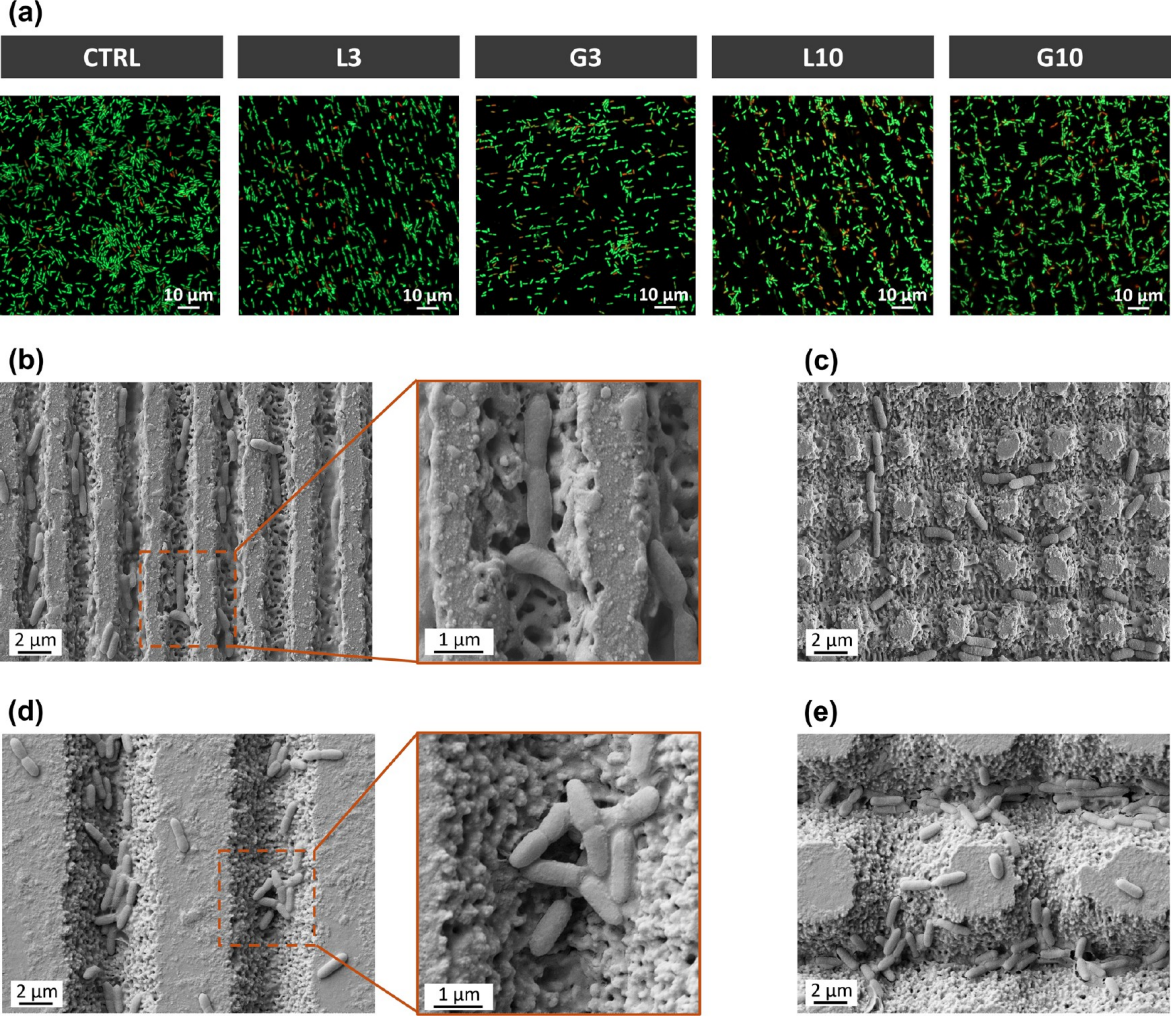
Results from the P. aeruginosa-adhesion assay. (a) Fluorescence CLSM images of the live and dead staining of P. aeruginosa after 4 h of incubation on the different surfaces. Live bacteria are stained with SYTO9 (green) and dead ones with propidium iodide (red). (b–e) FESEM micrographs of P. aeruginosa cultured on (b) L3, (c) G3, (d) L10, and (e) G10.

Interestingly, fluorescent images indicated that the bacteria were not randomly distributed on the surfaces but rather positioned according to the patterns. This feature was further analyzed by SEM. As evidenced in Figure 8b–e, the micropatterns restricted P. aeruginosa distribution: most of the bacteria were located within the laser valleys on the grid and linear structures, but only a few were found attached to the polished top surface areas of the patterns. Additionally, the distribution and orientation of P. aeruginosa differed significantly between the 3 and 10 μm periodicities. On L3 and G3, the narrow and shallow valleys confined the bacteria, physically forcing them to align in parallel to the grooves’ direction, probably due to the bacterial elongated shape (see magnification from Figure 8b and also Figure 8c). The small dimensions of the valleys, comparable to the size of the bacteria, also prevented the formation of large bacterial clusters within the grooves. On the contrary, on L10 and G10 patterns, the wider and deeper valleys enabled a greater bacterial accumulation, without any specific orientation, leading to the formation of larger agglomerations within the grooves (magnification from Figure 8d,e).

### 3.4 Cell–Bacterial Coculture

Having observed a greater antibacterial effect on the lower periodicity patterns, only 3 μm periodic linear (L3) and grid (G3) samples were selected to further study the viability of hMSCs in coculture with P. aeruginosa. To this end, a *postimplantation infection* coculture method was followed: the cells were first cultured for 24 h, and then P. aeruginosa were seeded on the surfaces and cultured for another 2 h.

The results of the coculture assay are presented in Figure 9. While the overall number of cells attaching to the surfaces did not seem to vary among the samples after the infection (Figure 9a), significant differences were clearly observed in terms of the spreading, morphology, and integrity of the hMSCs adhered to the different surfaces (Figure 9 and Figure 9c). More specifically, following the incubation with bacteria (i.e., *post implantation infection*), hMSCs morphology was drastically affected on the polished sample (CTRL), compared to the monoculture (MC) condition (i.e., cell incubation without bacteria). Indeed, the cells exhibited a smaller shape and a strongly disrupted morphology indicative of a cell necrotic state. These damaged cells are highlighted with dashed red circles in Figure 9c, and their disrupted morphology is clearly visible in the high-magnification fluorescence image. On the contrary, the cells adhered to both patterns (L3 and G3) preserved an optimal spreading and cytoskeletal elongation in the coculture condition, maintaining a morphology very similar to that observed in MC. This drastic difference in cell morphology was reflected in the reduced cell area on the CTRL sample compared to both micropatterns (Figure 9b).

**Figure 9:**
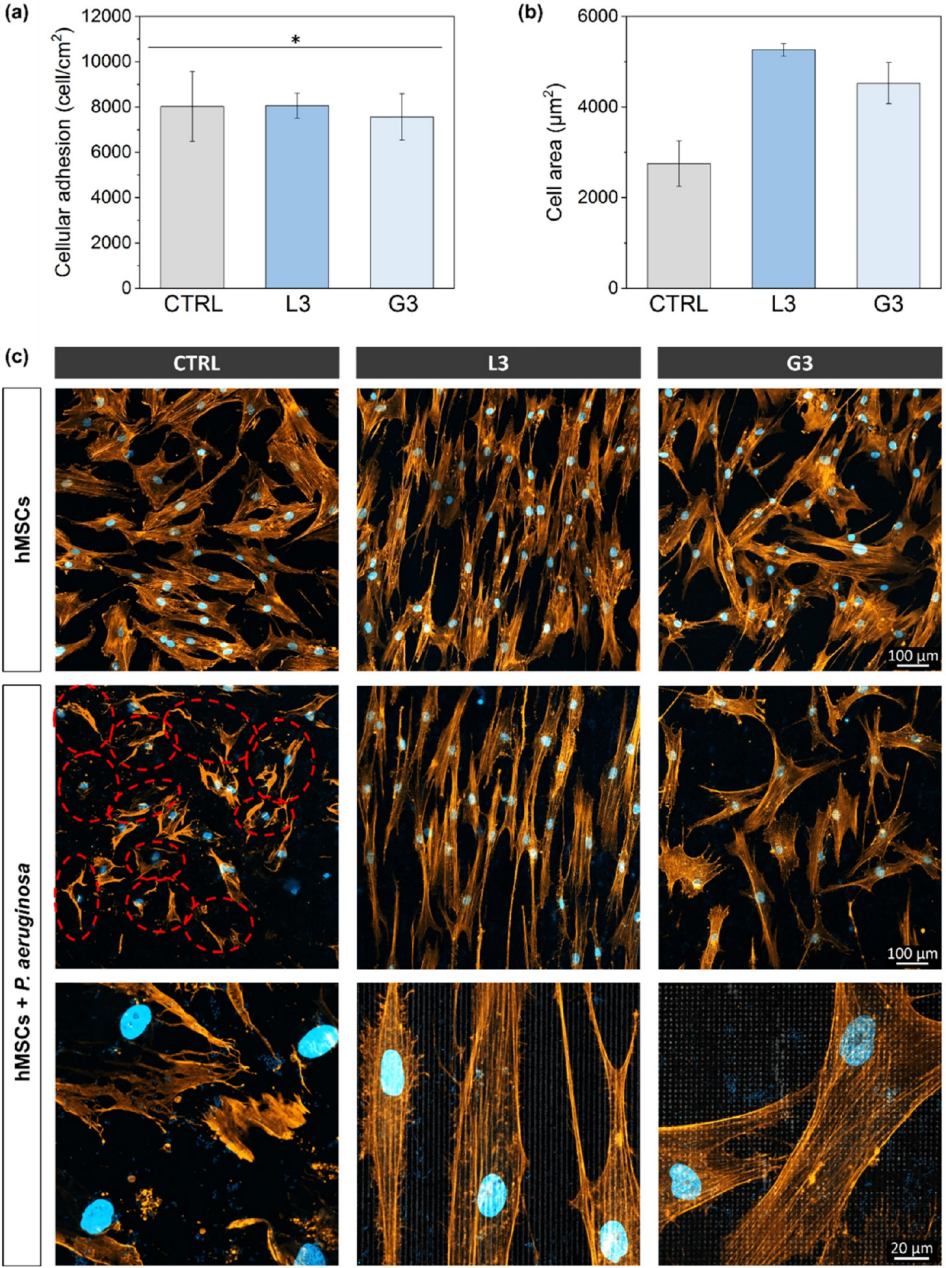
Result from the hMSCs coculture assay with P. aeruginosa, following a postimplantation 2-h coculture incubation procedure. (a) Cellular adhesion (cell/cm^2^) and (b) cell area (μm^2^). Group sharing the same symbol (*) show no significant difference (*p* < 0.05). (c) Representative fluorescence images of the coculture assay, with cell nuclei and bacteria stained with DAPI (blue) and actin filaments with phalloidin–Alexa Fluor 546 (orange). In CTRL, disrupted cytoskeletons are highlighted using dashed red circles.

To further quantify these observations, coculture cell integrity was measured based on actin staining from the fluorescence images (Table 4). The results strongly align with the morphological observations described above. Specifically, cell integrity was significantly lower in the CTRL samples (11%), confirming extensive cellular damage. In contrast, most of the cells adhered to the linear (L3) and grid (G3) patterns, maintaining high integrity (99% and 97%, respectively).

**Table 4:** Result from the Cell Integrity Quantification (%) of hMSCs Cocultured with P. aeruginosa Bacteria

	**CTRL**	**L3**	**G3**
**hMSCs integrity when cultured with** P. aeruginosa **(%)**	11 ± 11	99 ± 1^a^	97 ± 2^a^

a Indicates statistically significant differences with respect to CTRL (*p* < 0.05).

## 4 Discussion

Surface modification via USP-DLIP was proven to be an effective technique for generating well-defined, high-aspect-ratio, and reproducible micropatterns on 3Y-TZP surfaces. This method offers the possibility to tailor the morphology and topographic parameters by adjusting the optical setup and the laser processing parameters. In this study, USP-DLIP allowed us to create 3 and 10 μm linear (L3, L10) and grid (G3, G10) patterns with high precision, achieving periodicities very close to the target values. This precision in the structure fabrication is crucial for systematically investigating the influence of specific topographical features on cellular and bacterial responses.

The minimal surface and subsurface damage of the patterns (Figure 1b,c) highlights the precision of fs-laser processing, where material removal occurs primarily through evaporative ablation. In fact, this is a key advantage of fs-laser over nanosecond (ns) laser topographical modification when working with zirconia.^27^ In the fs regime, the ultrashort pulse duration (below the electron–phonon relaxation time) prevents heat transfer into the material, leading to evaporative ablation.^28^ In contrast, ns-lasers allow sufficient time for heat diffusion, and topography formation involves melting and resolidification that affects material integrity.^41^ This leads to significant differences in the surface quality and mechanical properties. Ns- lasers produce large molten traces, material pileups, and micrometric size surface and subsurface flaws,^30,32,41,42^ reducing the mechanical strength.^39,43,44^ In contrast, fs- lasers allow higher aspect ratio structuring with minimal nanometric size defects generation,^21,31,45^ preserving the mechanical properties.^39^

As expected, the average surface roughness (*Sa*) increased after the laser patterning, in line with other previous works.^21,41,43^ Furthermore, the topographical analysis revealed a strong dependence of the groove depth and width with the pattern periodicity (Table 2). For the 3 μm periodicity patterns (L3 and G3), the valleys were narrower and shallower, while for the 10 μm periodicity patterns (L10 and G10), both depth and width increased significantly. This variation is directly related to the laser processing parameters, mostly laser power, scan speed, and repetition rate, which were adjusted to create the L10 and G10 patterns. This proportional relationship between pattern size (i.e., width, depth) and laser processing parameters has been well-documented in previous studies.^21,30,39,41^

In addition to the microfeatures (i.e., lines and grids), a distinctive nanotopography was produced within the valleys because of complex laser-material interactions (Figure 1). The valleys of L3 and G3 exhibited periodic nanoripples resembling laser-induced periodic surface structures (LIPSS).^28^ This is consistent with previous studies on fs-laser-patterned zirconia, where these line-like periodic structures were formed with a spatial period slightly below the laser wavelength.^21,45–47^ LIPSS formation mechanism is very complex, as it involves various factors such as the laser’s energy, polarization, and wavelength interacting with the material’s surface.^28,48^ In contrast, L10 and G10 samples showed nanocavities within the grooves, likely resulting from localized boiling during high-energy laser irradiation, where the bursting bubbles leave behind these nanocavities.

The wettability results (Figure 2b) showed a strong dependence on the surface geometry. Linear patterns exhibited anisotropic wetting behavior, with higher contact angles perpendicular to the grooves due to the high energy barriers at valley edges that hinder droplet spreading, consistent with previous observations.^49,50^ This effect was more pronounced in L3, which exhibited stronger droplet pinning in the perpendicular direction compared to that in L10. In contrast, grid patterns showed isotropic wetting behavior, as the surface symmetry generated uniform energy barriers for droplets spreading. Pattern size also influenced wettability: Smaller structures (L3 and G3) exhibited higher water CAs, indicating stronger droplet pinning,^30,43,45,51^ while larger patterns (L10 and G10) showed lower CAs due to reduced pinning and enhanced droplet spreading. This size-dependent wetting behavior aligns with the findings by Ji et al.,^52^ who demonstrated that varying the depth and width of micropatterns could tune the wetting characteristics of surfaces. Chemical composition was also modified after surface irradiation. The increase in the C content can be attributed to surface carbonization after laser irradiation. Correspondingly, the percentages of *Y* decreased.

Cell attachment (i.e., number of cells) and spreading (i.e., cell area) were enhanced on the laser-patterned surfaces (L3, G3, L10, and G10) compared to the polished surface (CTRL), as the micro- and nanostructures provide anchoring points required for the cells to adequately attach and spread (Figures 3 and 4e–g). Accordingly, improved cell adhesion and spreading have been previously evidenced in laser microgrooved zirconia.^21,32,34^ Nevertheless, hMSCs attachment and spreading were similar across all patterns, suggesting this type of cell response was independent of each of the specific pattern’s characteristics. The changes in wettability were also expected to influence the hMSCs response; however, the variations in water contact angle did not seem to have a marked effect in our study. Indeed, cell adhesion was increased on all patterns, irrespective of showing similar (G10), lower (L10, parallel), or higher (L3 perpendicular, G3) contact angle values in comparison with CTRL samples.

Furthermore, vinculin staining highlighted the capacity of laser-induced nanotopography to promote strong cell-surface interactions (Figure 5). The cells anchor to the extracellular matrix (ECM) via integrin proteins, which then link to the actin filaments from the cell via intracellular multiprotein complexes (e.g., vinculin, talin, etc.).^53^ These large adhesion complexes, known as focal adhesion (FAs), play a crucial role in mechanosensing, triggering intracellular signaling pathways that regulate cytoskeleton reorganization and key cellular functions such as adhesion, migration, and differentiation.^15,20,53,54^ In this respect, it is well-known that nanoscale topographical features can influence integrin clustering and therefore promote FAs formation.^53–55^ In our study, nascent FAs were generated on the laser-structured samples. The vinculin plaques were primarily located at the cell periphery and within the grooves, suggesting that the nanofeatures from the valleys facilitated integrin clustering and FA assembly. Additionally, FESEM inspection corroborated that cells interact with the nanoroughness within the valleys through the extension of filopodia from the cell body (Figure 4).

In addition, our study also revealed a strong dependence on cell morphology and alignment with the pattern’s structure type (Figures 3d,e and 4), highlighting the importance of microtopography in guiding cellular morphological changes. On the linear patterns (L3 and L10) the cells exhibited an elongated shape with protrusions growing and aligned along the grooves, consistent with previous studies.^21,47^ These linear structures provided directional cues (i.e., physical barriers) that promoted cytoskeletal reorganization, guiding cells toward a directional-elongated shape. In a previous work, Leclech et al.^22^ showed a consistent trend toward enhanced elongation and alignment on narrower and deeper grooves. Nevertheless, in our study, both linear patterns produced similar outcomes regardless of the groove size. On the contrary, on the grid patterns (G3 and G10) the size of the grid played a more significant role. Larger grids (G10) guided cells to grow and align along both grid directions, while smaller grids (G3) provided less prominent directional cues, resulting in widespread and less aligned cells.

The cell migration assay demonstrated significant improvements in hMSCs motility and wound healing properties on all patterned surfaces (Figure 6). This enhanced migration is given by the micropatterns guiding cell orientation and the formation of nascent FAs.^56^ While previous studies emphasized cell elongation as the main factor in topography-induced migration,^21^ our results suggest that cell alignment (i.e., topography-mediated cell morphology) plays a more critical role. This is evidenced by the similar migration lengths and wound closure achieved on both linear (elongated cells) and grid (nonelongated cells) patterns. Notably, the greater migration length achieved along L10 and G10 indicates that cells migrate more efficiently when provided with wider channels.^57^ Furthermore, the linear patterns promoted anisotropic migration (strongly preferential along one axis), while grid patterns facilitated cell migration in both directions (Figure 6b). Besides microtopography, the nanotopography of the grooves plays also a significant role in cell migration by promoting the formation of FAs, which provide dynamic anchoring points for faster movements.^47,54^ Interestingly, small or nascent FAs, like the ones observed in our study, have shown greater effects on cell movement compared to mature FAs, as the former undergo a more rapid assembly disassembly cycle involved in cell migration.^58^

Besides influencing cell response, surface properties are known to be determinant factors influencing bacterial adhesion as well.^25^ Bacteria adhere to surfaces through specialized appendages (pili, fimbriae) and/or protein complexes (adhesins), with this adhesion being modulated by external factors including surface wettability, chemistry, and topography.^59^ Unlike mammalian cells, which can adapt their cytoskeleton to micro and nanotopography due to their size (10–100 μm) and flexibility,^60^ bacteria are smaller (1–2 μm) and rigid due to their cell wall structure.^61^ This rigidity makes bacteria particularly susceptible to topographical features smaller than their size (nano- or submicron). While laser texturing has been widely used to create antibacterial surfaces on different materials,^36–38,62^ few studies have explored this effect on laser-patterned zirconia.^32,35^ In other materials, the antibacterial properties are often associated with nanosized (200–800 nm) spikes, holes, ripples, or LIPSS, which can inhibit bacterial adhesion across various strains. In our study, however, despite the presence of nanofeatures within the valleys, the bacterial reduction was linked to microstructural features.

In fact, the distinct responses obtained between P. aeruginosa and S. aureus revealed a complex interplay among surface topography, wettability, and bacterial strain. P. aeruginosa, which is a motile rod-shaped bacterium, showed reduced attachment on all of the patterns compared to the polished surface, primarily due to topographical effects rather than wettability changes. As evidenced by the uneven distribution of the bacteria (Figure 8b–e), the microtopography restricted bacterial proliferation and motility by trapping the bacteria within the valleys of the linear and grid patterns.^63^ This mechanical confinement is consistent with previous findings indicating that such a mechanism can prevent biofilm formation by limiting bacterial interactions and quorum sensing.^63–65^ Moreover, Chang et al.^66^ demonstrated a reduction in P. aeruginosa motility in structures with a width of 2–8 μm. Furthermore, the pattern size influenced bacterial retention. The smaller patterns (L3 and G3) reduced colonization by 25%, aligning bacteria within grooves that matched their diameter. Larger patterns (L10 and G10) also reduced adhesion but to a lesser extent as the bacteria formed larger clusters within the wider and deeper grooves.

For S. aureus, a nonmotile round-shaped bacterium, only the low periodicity patterns (L3 and G3) reduced their adhesion (Figure 7a). Unlike P. aeruginosa, S. aureus adhered homogeneously to both the valleys and polished zones, suggesting a different antibacterial mechanism. We propose that the bacterial colonization reduction on L3 and G3 is due to the small-size grooves providing physical obstacles that hinder the formation of S. aureus aggregation.^62,67^ To further investigate this, we estimated the groove volume for each microtopography (Supporting Information S4) to assess the available space for bacterial cluster formation. The grooves in L3 and G3 have approximately 10 times less volume than those in L10 and G10, restricting more of the space for bacterial aggregations (Figure 7b) and consequently reducing colonization. In contrast, the wider and deeper grooves in L10 and G10 allowed S. aureus to form larger agglomeration, similar to the CTRL (Figure 7b), and thus, they did not inhibit bacterial-surface colonization. This microtopography-mediated inhibition of S. aureus colonization aligns with previous studies, where microtopographies with dimensions similar to L3 and G3 patterns effectively blocked bacterial cluster formation and reduce biofilm development.^67,68^

In summary, while all patterns displayed similar cell-enhanced properties, the low periodicity patterns, matching the bacterial size more closely, were more effective in reducing colonization through trapping (P. aeruginosa) and other repelling mechanisms (S. aureus). Indeed, the coculture assay confirmed that L3 and G3 successfully combined cell-instructive and antibacterial properties (Figure 9). In our postinfective setting, these patterns preserved optimal cell attachment and morphology in the presence of bacteria, allowing cells to “win” the race for the surface in such a competitive scenario. In contrast, cell adhesion was strongly affected by the presence of bacteria on the polished control surfaces, resulting in cell necrosis. Therefore, the low periodicity patterns produced in this study hold potential to fine-tune cell responses, even in challenging postoperative infections.

## 5 Conclusions

Our study demonstrates that USP-DLIP is a versatile technique for generating well-defined micropatterns on zirconia surfaces with enhanced cell responses and antibacterial properties. The fs-laser DLIP method allowed us to generate high-aspect-ratio linear and grid structures with minimal defects and nanoroughness, allowing for a detailed exploration of how specific topographies influence cellular and bacterial behaviors.

Cells responded to the micropatterns by adjusting their attachment, morphology, and spreading, leading to enhanced adhesion and migration. The nanotopography provided stronger cell anchoring, while the micropatterns guided the cell morphology and accelerated wound healing through enhanced migration. Additionally, the laser-patterned surfaces demonstrated antibacterial properties against P. aeruginosa and S. aureus. The smaller patterns (L3 and G3) were particularly effective in reducing bacterial colonization through mechanical trapping (P. aeruginosa) and disruption of bacterial aggregations (S. aureus).

Notably, L3 and G3 exhibited the best combination of cell-instructive and antibacterial properties. Their ability to reduce bacterial colonization, coupled with their capacity to promote cell adhesion, spreading, and migration, makes these patterns ideal candidates for developing more durable and biocompatible zirconia dental implants. Moreover, we believe our findings may prove useful for designing a wider range of materials and also for distinct medical applications, e.g., orthopedic materials. In this regard, future research using laser patterning should focus on enhancing the antibacterial properties through reduced pattern size, by implementing additional nanotopographies, or by means of functionalization with antibacterial biomolecules.

## Supplementary Material



## Notes

Topographical parameters of linear and groove patterns, confocal laser scanning microscopy (CLSM) imaging of L3, G3, L10, and G10, surface wettability, S. aureus inhibition mechanism (PDF)
