# Supplementary material for: Tailoring Cell Behavior and Antibacterial Properties on Zirconia Biomaterials through Femtosecond Laser-Induced Micropatterns and Nanotopography
Source: ACS Appl Mater Interfaces. 2025 May 10;17(20):29082–99. doi: 10.1021/acsami.4c22433 (PMC12133025; doi:10.1021/acsami.4c22433)
Supplement: Supplementary file 1 [file am4c22433_si_001.pdf]

Supporting information

Tailoring cell behavior and antibacterial properties on zirconia biomaterials through femtosecond laser-induced micropatterns and nanotopography

N. Garcia-de-Albeniz [1,2,3], D.W. Müller [4], F. Mücklich [4], M.-P. Ginebra [2,3,5,6], E. Jimenez-Piqué [1,3,*], C. Mas-Moruno [2,3,5,*]

[1] *Center for Structural Integrity, Reliability and Micromechanics of Materials (CIEFMA), Department of Materials Science and Engineering, Universitat Politècnica de Catalunya. BarcelonaTech (UPC), Av. Eduard Maristany, 16, 08019 Barcelona, Spain*

[2] *Biomaterials, Biomechanics and Tissue Engineering Group (BBT), Department of Materials Science and Engineering, Universitat Politècnica de Catalunya. BarcelonaTech (UPC), Av. Eduard Maristany, 16, 08019 Barcelona, Spain*

[3] *Barcelona Research Center in Multiscale Science and Engineering, Universitat Politècnica de Catalunya. BarcelonaTech (UPC), Av. Eduard Maristany, 16, 08019 Barcelona, Spain*

[4] *Functional Materials, Department of Materials Science and Engineering, Saarland University, 66123 Saarbrücken, Germany*

[5] *Centro de Investigación Biomédica en Red—Bioingeniería, Biomedicina y Nanomedicina (CIBER-BBN), Instituto de Salud Carlos III, 28029 Madrid, Spain*

[6] *Institute for Bioengineering of Catalonia (IBEC), Barcelona Institute of Science and Technology, 08028 Barcelona, Spain*

** Corresponding authors, e-mail: emilio.jimenez@upc.edu (E. Jiménez-Piqué) and carles.mas.moruno@upc.edu (C. Mas-Moruno)*

S1. Topographical parameters

A schematic description of the topographical features characterized by CLSM.

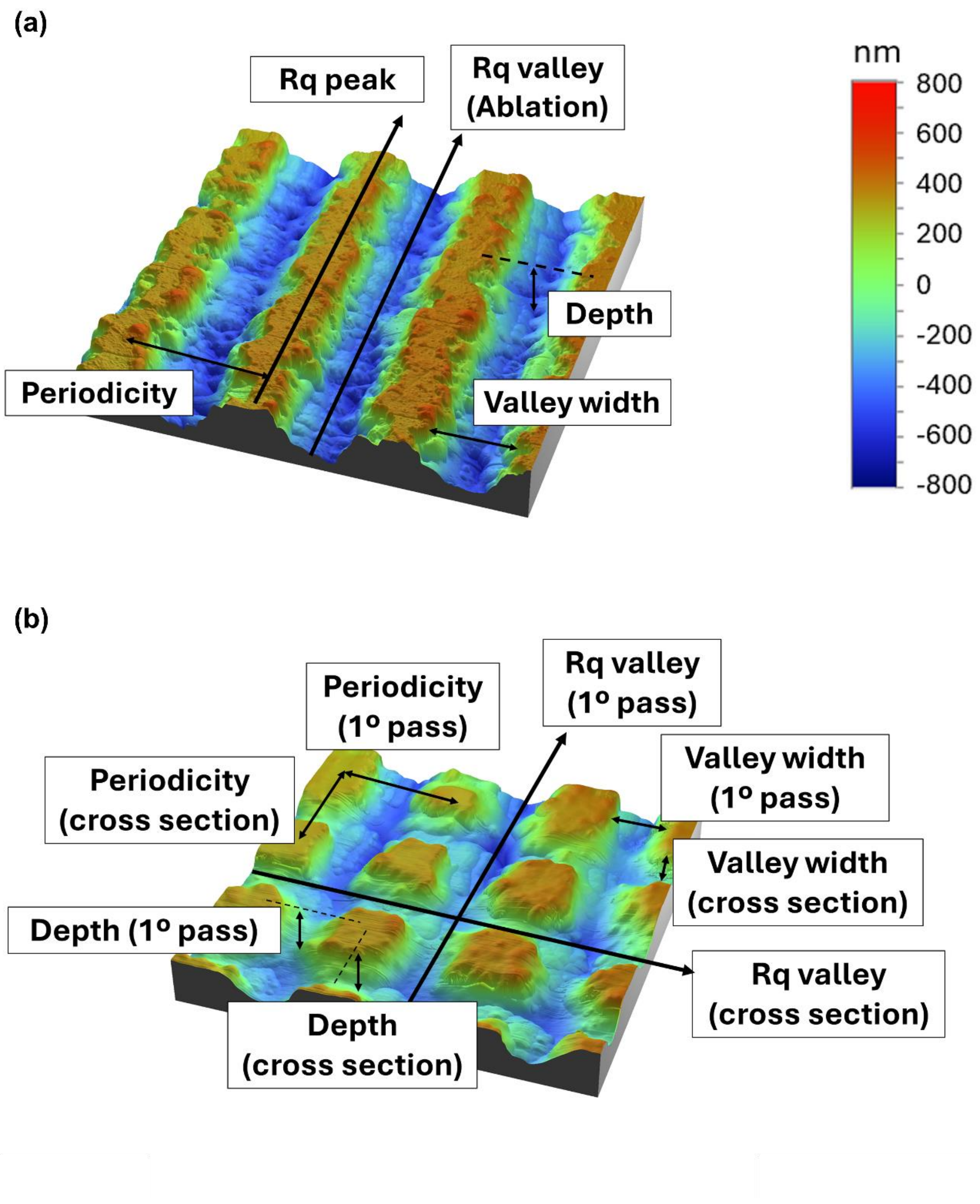

Figure S1. 1. Description of the topographical features characterized in the (a) linear and (b) grid patterns.

S2. 3D topography images and profiles of micropatterns

The 3D topographies and profiles obtained from the CLSM are included below.

L3 sample

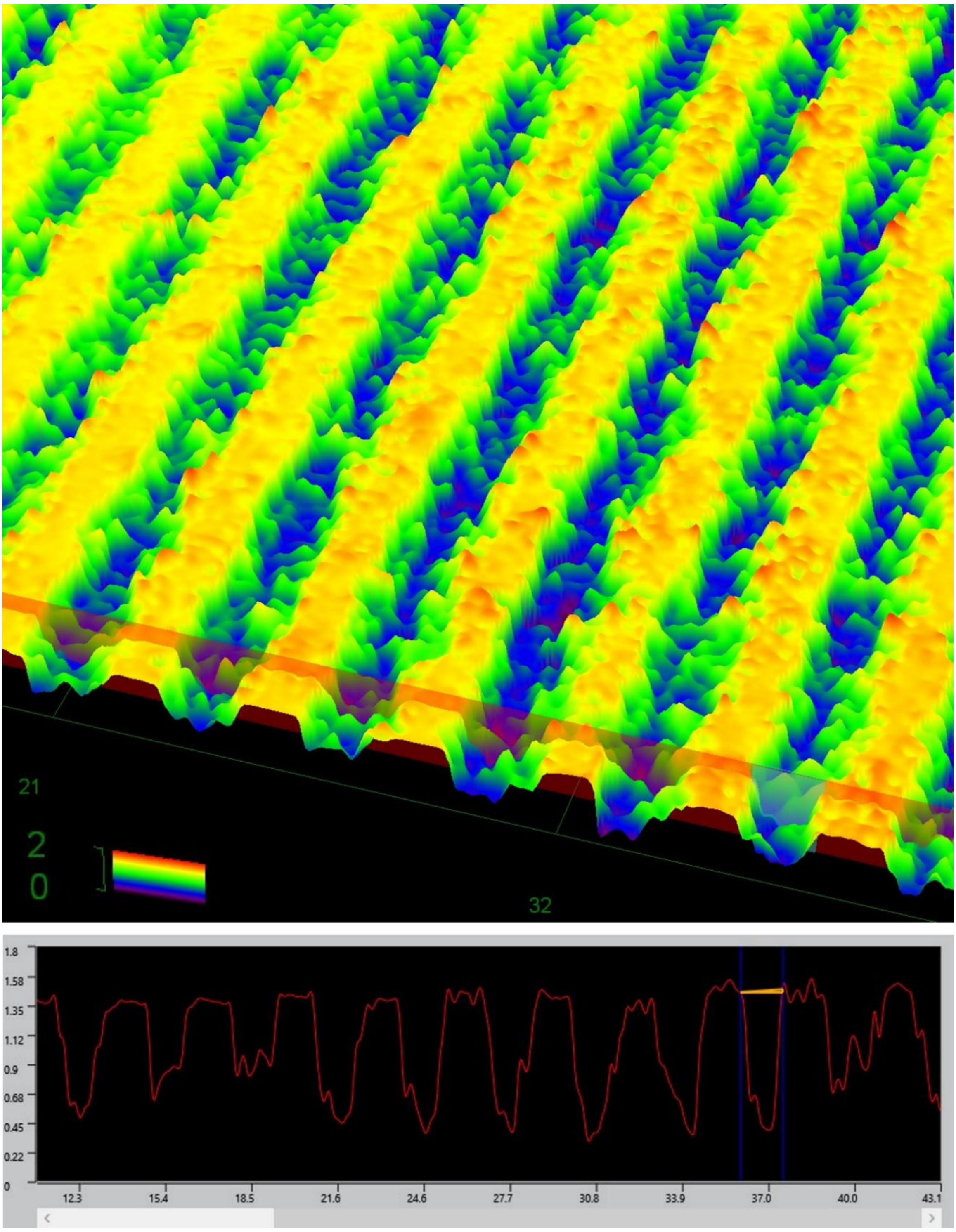

Figure S2. 1. 3D color-mapping and topography profile of the L3 patterns obtained using CLSM. Distances and scale bars are in micrometers.

G3 sample

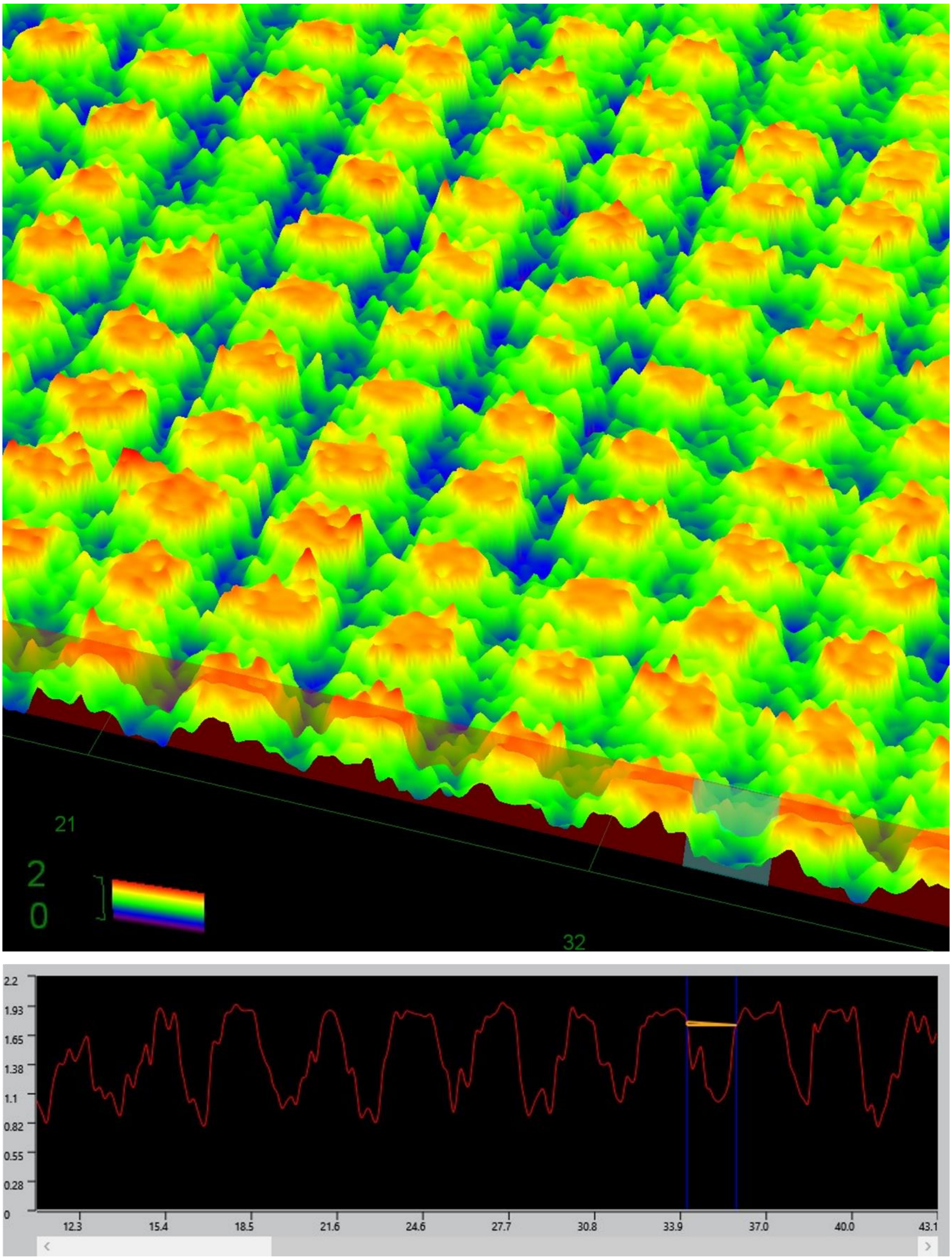

Figure S2. 2. 3D color-mapping and topography profile of the G3 patterns obtained using CLSM. Distances and scale bars are in micrometers.

L10 sample

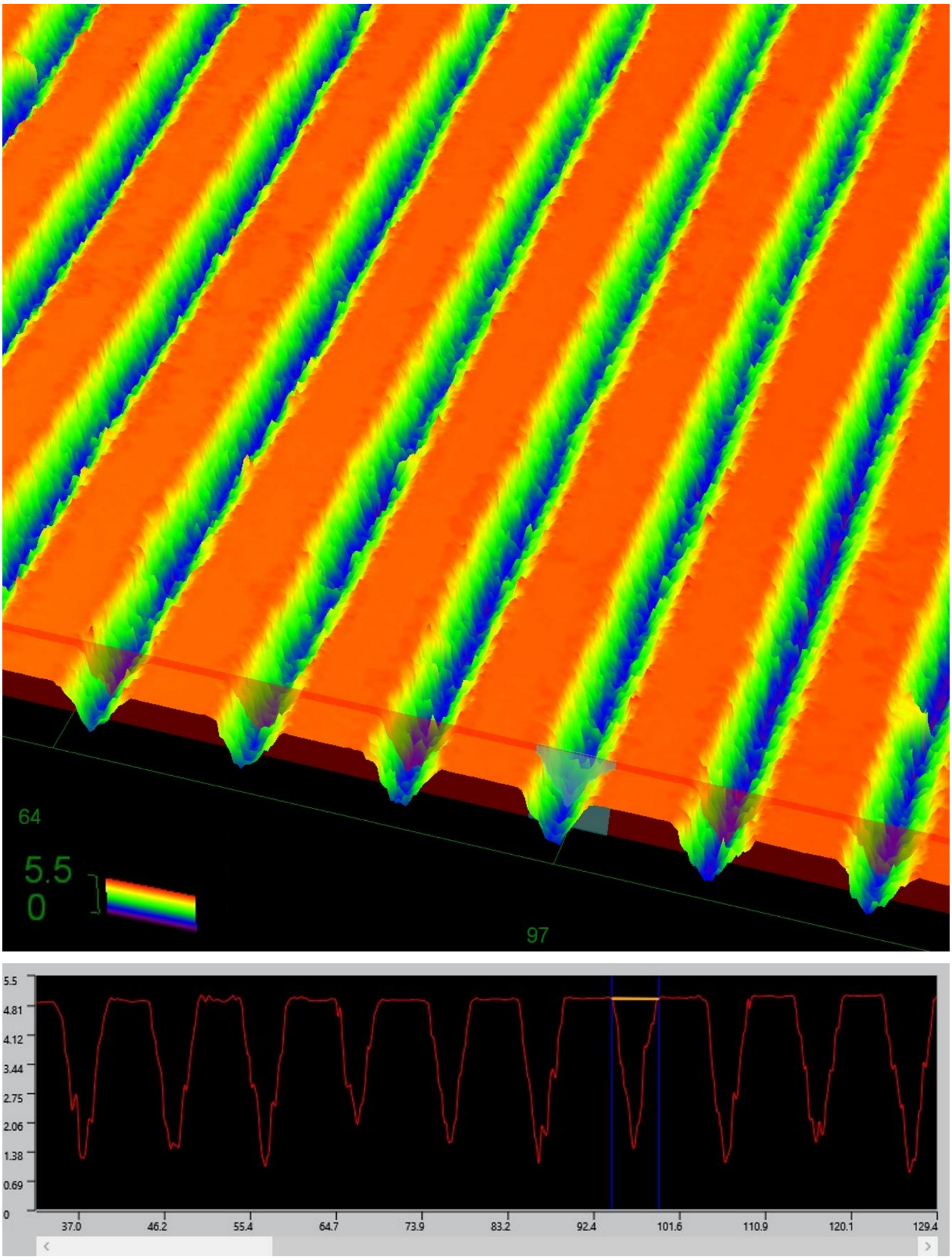

Figure S2. 3. 3D color-mapping and topography profile of the L10 patterns obtained using CLSM. Distances and scale bars are in micrometers.

G10 sample

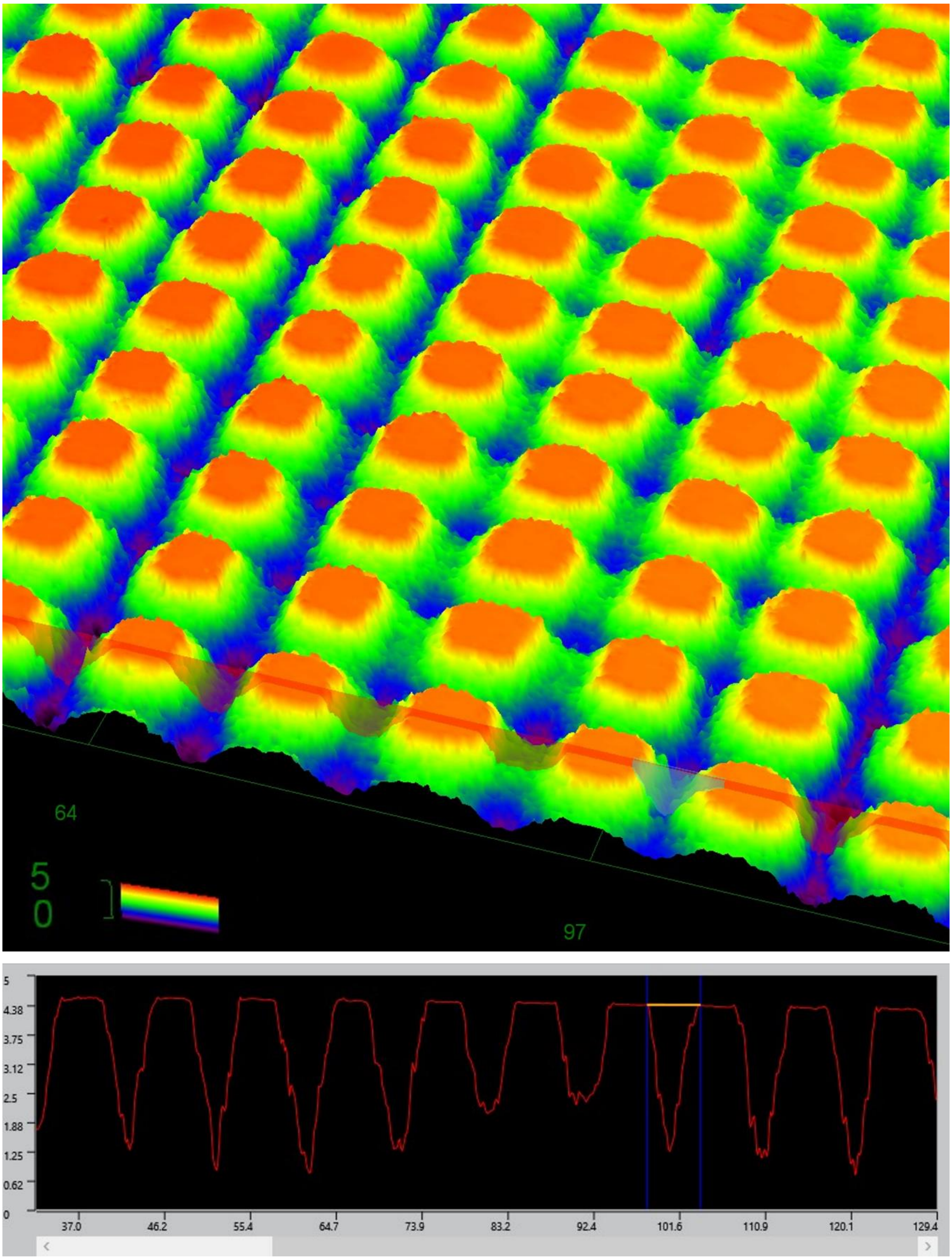

Figure S2. 4. 3D color-mapping and topography profile of the G10 patterns obtained using CLSM. Distances and scale bars are in micrometers.

S3. Surface wettability

The Wenzel approach was used to calculate the intrinsic contact angle (θ) of the patterned surfaces. As detailed in the experimental procedure, the water contact angle (CA) was measured in both pattern directions.

Table S3.1 summarizes the wettability results, including the measured contact angles (θ_m), intrinsic contact angle (θ), and the surface anisotropy calculation (Δθ).

Samples	S_{dr}	r	$\theta_{=m}$	$\theta_{\perp m}$	$\theta_{=}$	$\theta_{\perp}$	$\Delta\theta$
**CTRL**	-	-	55.6 ± 2.2		-		-
**L3**	65.4	1.65	51.0 ± 5.4	96.5 ± 1.4	67.7 ± 2.8	94.9 ± 0.9	27.2
**G3**	47.6	1.48	74.8 ± 1.3	80.2 ± 5.1	74.8 ± 1.3	79.8 ± 0.9	3.6
**L10**	74.2	1.74	37.6 ± 5.1	60.8 ± 5.6	63.0 ± 2.0	73.8 ± 2.9	10.8
**G10**	78.7	1.79	48.2 ± 2.6	53.8 ± 3.7	68.2 ± 1.2	70.8 ± 1.8	2.6

Table S3.1. Wettability parameters of CTRL and patterned samples. Developed surface area (S_{dr}) and roughness factor (r) of the patterns, measured CA in the parallel ($\theta_{=m}$) and perpendicular ($\theta_{\perp m}$) directions, intrinsic CA in the parallel ($\theta_{=}$) and perpendicular ($\theta_{\perp}$) directions, and wettability anisotropy ($\Delta\theta = |\theta_{\perp} - \theta_{=}|$).

S4. *S. aureus* inhibition mechanism

To further explain the *S. aureus* bacterial inhibition mechanism provided by our microtopographies, we estimated the volume of the grooves in each micropattern to approximate the number of bacteria that could fit inside (**Table S4.1**).

Figure S4.1 shows a schematic representation of the groove volume calculation, where *a* represents a fixed groove length (5 µm for all samples), and *b* and *c* correspond to the groove width and depth, respectively, which vary between samples. Since the grooves have a triangular profile, this shape was also considered in the volume calculation.

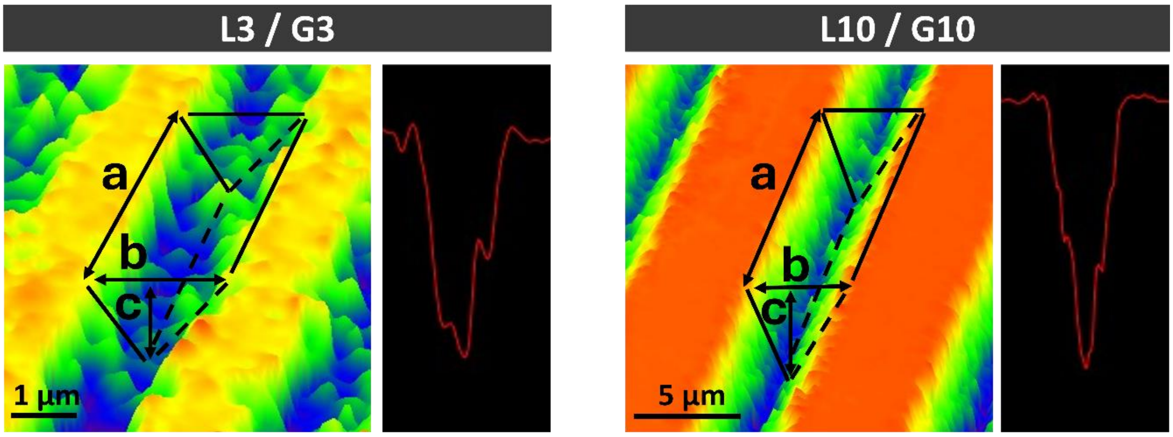

Figure S4. 1. Schematic description of the distances used for the groove volume calculation in L3, G3, L10 and G10. In the images, a is the groove length (5 µm for all samples), and b and c correspond to the groove width and depth, respectively.

Sample	a (µm)	b (µm)	c (µm)	Groove volume (μm^3)	Number of *S. aureus* per calculated groove volume
**L3**	5	1.45	1.20	4.35	~ 16
**G3**	5	1.49	1.07	3.96	~ 15
**L10**	5	5.09	3.16	40.21	~ 150
**G10**	5	5.25	3.15	41.34	~ 154

Table S4. 1. Calculation of the groove volume for each micropattern and the estimated number of S. aureus bacteria that fit within that volume.

Based on these calculations, the grooves in high periodicity patterns (L10, G10) have approximately 10 times more volume than those in L3 and G3, allowing more bacteria to accommodate inside. Following this, we have estimated the number of *S. aureus* that can fit into these grooves considering a bacterial volume of 0.27 μm^3 (calculated from a 0.8 µm diameter for *S. aureus*). The results showed that, for a given groove length, only about 15 bacteria can fit in L3 and G3, while nearly 150 bacteria can fit in L10 and G10. This greater bacterial accommodation in L10 and G10 facilitates the formation of larger *S. aureus* clusters, whereas in the small patterns bacteria are unable to form big agglomerations.
